# Translational lipidomics in glaucoma: mechanisms, biomarkers, and therapeutic horizons

**DOI:** 10.3389/fphar.2026.1921097

**Published:** 2026-10-26

**Authors:** Abu Bakar Butt, Michael Balas, Jeremy M. Sivak, David J. Mathew

**Affiliations:** 1 Schulich School of Medicine & Dentistry, Western University, London, ON, Canada; 2 Department of Ophthalmology & Vision Sciences, University of Toronto, Toronto, ON, Canada; 3 Donald K. Johnson Eye Institute, University Health Network, Toronto, ON, Canada

**Keywords:** biomarker, glaucoma, IOP (intraocular pressure), lipidomics, therapeutic

## Abstract

Glaucoma is a multifactorial optic neuropathy. Intraocular pressure (IOP), aging, vascular dysregulation, oxidative stress, mitochondrial failure, and chronic neuroinflammation converge on retinal ganglion cell (RGC) degeneration and optic nerve damage. Lipid mediators are central to this biology, linking membrane remodeling to aqueous humor (AH) dynamics, glial activation, vascular tone, extracellular matrix turnover, and neuronal survival. Lipidomics has widened the field beyond prostaglandin pharmacology to include oxylipins, lysophospholipids, sphingolipids, endocannabinoids, and specialized pro-resolving mediators (SPMs). Human studies in AH, tears, and plasma report reproducible perturbations in arachidonic acid (AA) metabolism, oxidized linoleate products, phosphatidylcholines, lysophosphatidylcholines, sphingomyelins, and ceramides; experimental models further implicate ceramide accumulation, phospholipase A2 activation, cyclooxygenase signaling, endocannabinoid tone, and failed inflammation resolution in retinal injury. Recent work also shows that latanoprost itself induces an aqueous-humor AA/LXA_4_ circuit in glaucoma patients, indicating that established prostaglandin therapy may engage endogenous resolution pathways and complicate the interpretation of treated-cohort lipidomic studies. This review covers the mechanistic roles of lipid mediators in IOP regulation, TM dysfunction, neuroinflammation, and neurodegeneration; the emerging biomarker literature in AH, tears, and plasma; and potential therapeutic directions, including soluble epoxide hydrolase (sEH) inhibition, sphingolipid modulation, endocannabinoid augmentation, and SPM-based neuroprotection. We conclude with the main barriers to translation: small ocular sample volumes, treatment confounding, limited longitudinal data, and the need for disease-subtype stratification.

## 1 Introduction

Glaucoma comprises a group of chronic progressive optic neuropathies characterized by structural damage to the optic nerve head and corresponding visual field loss (Coleman, 1999; Weinreb et al., 2014; Weinreb and Khaw, 2004; Leske, 2007). The disease is clinically heterogeneous, encompassing primary open-angle glaucoma (POAG), normal-tension glaucoma, pseudoexfoliation glaucoma (PEXG), primary angle-closure glaucoma, and multiple secondary glaucomas. Despite this heterogeneity, a common endpoint is degeneration of retinal ganglion cells (RGCs), including their somata, axons, and synaptic circuitry. Elevated intraocular pressure (IOP) remains the strongest modifiable risk factor, and lowering IOP is the foundation of all current treatments. Yet IOP alone does not fully explain disease onset, pace of progression, or treatment failure. Many patients with elevated IOP never develop glaucoma, while others progress despite statistically normal pressures, or after apparently adequate pressure reduction (Weinreb et al., 2014; Weinreb and Khaw, 2004; Vohra et al., 2013; Grieshaber et al., 2007). This clinical reality has shifted attention towards additional mechanisms, especially neuroinflammation, mitochondrial dysfunction, vascular insufficiency, oxidative injury, and age-related failure of tissue resilience (Vohra et al., 2013; Grieshaber et al., 2007; Casson, 2006; Li et al., 2017; Mirzaei et al., 2017). Experimental evidence further indicates that the temporal pattern of pressure exposure influences the underlying tissue response: gradual, sustained ocular hypertension produces optic-nerve cytokine and microglial signatures distinct from acute pressure elevation, despite comparable retinal ganglion cell loss (Brust et al., 2008).

Lipids are increasingly relevant to this broader framework. Historically, glaucoma therapeutics already provided one powerful example of their clinical value: prostaglandin analogues became the most successful topical drug class for chronic IOP lowering because they harness endogenous lipid signaling pathways to enhance aqueous outflow (Doucette and Walter, 2017; Lütjen-Drecoll and Tamm, 1988; Schachtschabel et al., 2000; Schmidl et al., 2015; Mathew and Sivak, 2024). A 2024 review by Mathew and Sivak synthesized emerging evidence linking lipid signaling to glaucoma pathophysiology, highlighting the potential roles of bioactive lipids in IOP homeostasis, inflammatory resolution, extracellular matrix remodeling, and neuronal survival (Mathew and Sivak, 2024). Because ocular tissues are highly compartmentalized and enriched in polyunsaturated fatty acids, the eye is especially susceptible to shifts in lipid metabolism under stress (Kolko et al., 2023; Langbøl et al., 2020). The spatial distribution of these lipid mediator pathways across anterior-segment, posterior-segment, and systemic compartments, together with established clinical targets, is summarized in Figure 1. Modern mass spectrometry platforms can profile hundreds to thousands of lipids from small ocular samples, allowing investigators to interrogate aqueous humor (AH), tear fluid, trabecular meshwork (TM), retina, and plasma in relation to glaucoma phenotypes (Umeno et al., 2019; Myer et al., 2020; Buisset et al., 2019; Ghosh and Nishtala, 2017; Aljohani et al., 2013; Edwards et al., 2020). Complementary AH proteomics has identified stage-associated immune signatures and nominated TRPC6 as a candidate marker associated with POAG severity, illustrating the value of integrating lipidomic and proteomic layers (Zhou et al., 2026). Glaucoma-associated lipid changes reflect coordinated remodeling of eicosanoid pathways, phospholipid turnover, sphingolipid balance, and endocannabinoid signaling (Mathew and Sivak, 2024). Some changes contribute directly to tissue damage; others are adaptive or compensatory. Distinguishing the two matters for translation, because biomarkers, mechanistic targets, and therapeutic agents need not come from the same branch of the network.

**FIGURE 1:**
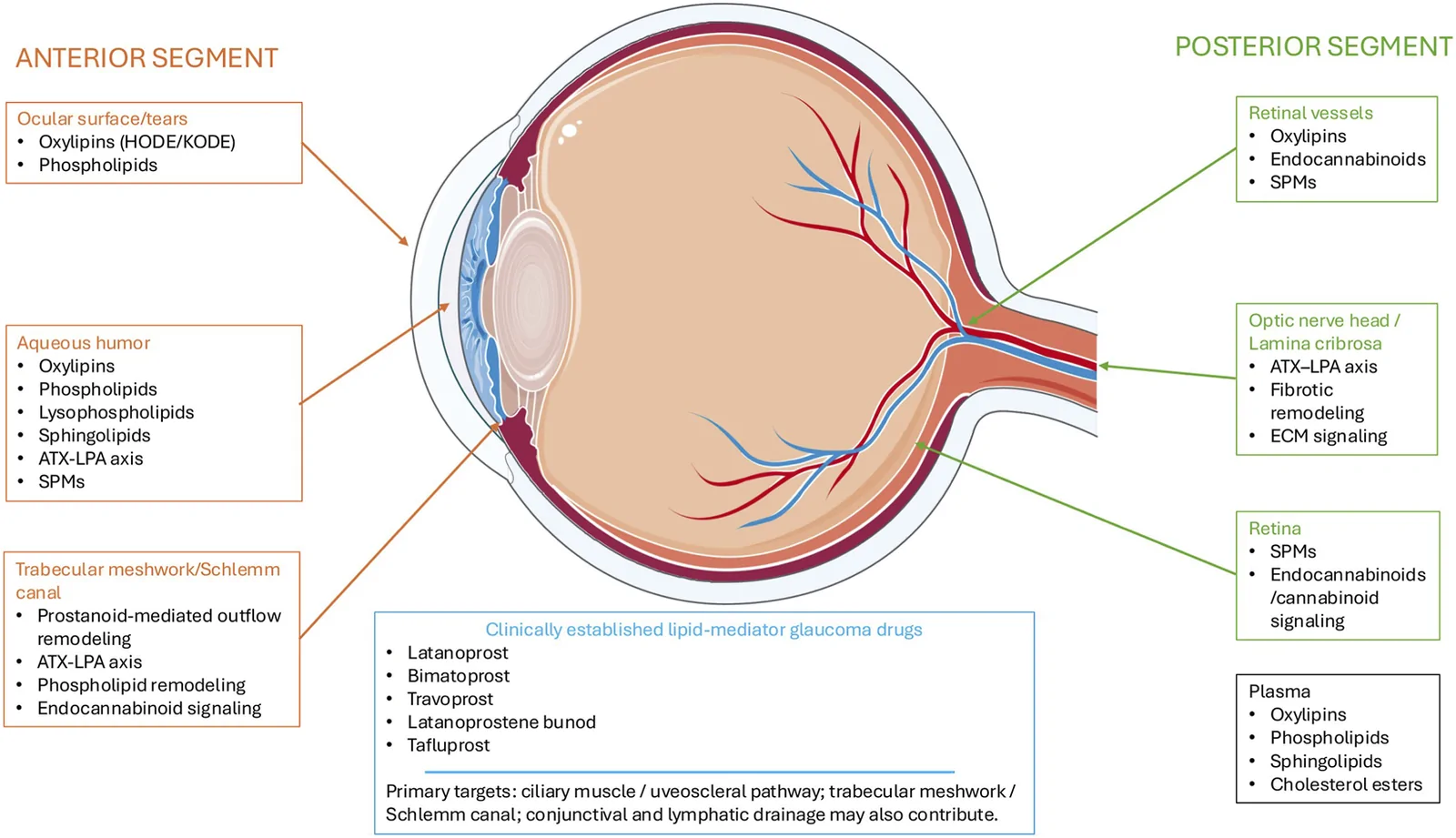
Schematic overview of anterior-segment, posterior-segment, and systemic lipid mediator pathways discussed in this review. Lipid classes listed in each compartment represent reported biomarker signals, proposed mechanistic mediators, or candidate therapeutic targets, depending on the available evidence. Aqueous humor, tear, and plasma compartments primarily reflect lipidomic biomarker data, whereas trabecular meshwork, Schlemm canal, ciliary muscle, retina, optic nerve head, and lamina cribrosa denote anatomical sites where lipid mediators may influence aqueous outflow, extracellular matrix remodeling, neuroinflammation, vascular regulation, or retinal ganglion cell injury. The drug box highlights clinically established lipid-mediator therapies, principally prostanoid-based intraocular pressure–lowering agents. Emerging pathways, including ATX–LPA signaling, sphingolipid remodeling, endocannabinoid signaling, sEH-related oxylipin metabolism, PAF-related lipids, and specialized pro-resolving mediators, remain investigational; clinical and mechanistic evidence for each is discussed in the text. Footnote: The plasma compartment is shown without color coding to reflect its systemic rather than ocular origin. Adapted from “Servier–Drawing Eye lateral view–no labels” by Servier Medical Art, via AnatomyTOOL.org. License: CC BY 4.0.

In the retina and optic nerve, astrocyte-derived LXA_4_/LXB_4_ and DHA-derived neuroprotectin D1 protect RGCs after injury, implicating pro-resolving lipid pathways in neuroprotection (Qin et al., 2008; Livne-Bar et al., 2017). In anterior-segment tissues, the autotaxin–LPA axis has been linked to elevated IOP, aqueous outflow resistance, fibrogenic activity, and downstream Rho kinase signaling; emerging work also implicates LPA-axis dysregulation in lamina cribrosa fibrotic remodeling (Igarashi et al., 2020; O’Regan et al., 2021). Mechanosensitive pathways further connect pressure stress to lipid mediator release, as TRPV4 activation in trabecular meshwork cells promotes endogenous prostaglandin production and IOP lowering (Uchida et al., 2021).

Three developments make a synthesis useful now: the mechanistic literature has matured into integrative models linking pressure stress with lipid-mediated inflammation and neurodegeneration; biomarker studies have nominated candidate lipid signatures for disease detection and subtyping; and therapeutic opportunities are expanding beyond prostanoid pharmacology toward endocannabinoid modulation, sEH inhibition, ceramide targeting, and pro-resolving lipid replacement. This review synthesizes those developments, with attention to what is established, what remains uncertain, and what is most likely to impact clinical practice.

### 1.1 Literature search strategy

A narrative approach was adopted given the breadth and heterogeneity of the lipidomics literature spanning mechanistic, biomarker, and therapeutic domains; a systematic synthesis was not attempted, as the aim was to integrate mechanistic, biomarker, and therapeutic literature into a cohesive translational narrative rather than to quantify effect sizes or appraise study-level risk of bias across a homogeneous outcome set. PubMed/MEDLINE, Embase, and Google Scholar were searched from inception to 10 April 2026 using combinations of terms including “glaucoma,” “lipidomics,” “aqueous humor,” “eicosanoids,” “sphingolipids,” “endocannabinoids,” “prostaglandin analogue,” and “specialized pro-resolving mediators.” Reference lists of key reviews and primary studies were hand-searched for additional relevant articles. Preference was given to peer-reviewed original research and reviews published in English; articles were included based on relevance to lipid signaling in glaucoma pathophysiology, biomarker discovery, or therapeutic development, with final selection guided by the authors’ clinical and mechanistic judgment rather than predefined inclusion/exclusion criteria.

## 2 Lipid mediator systems relevant to glaucoma

### 2.1 Oxylipins and eicosanoids

Oxylipins are oxygenated derivatives of polyunsaturated fatty acids generated through cyclooxygenase (COX), lipoxygenase (LOX), cytochrome P450 (CYP), and non-enzymatic oxidation pathways (Lima et al., 2012; Gabbs et al., 2015). Among them, AA-derived prostaglandins have the longest history in ophthalmology (Mathew and Sivak, 2024). FP receptor agonists such as latanoprost, travoprost, tafluprost, and bimatoprost revolutionized glaucoma treatment by enhancing uveoscleral outflow, and depending on the agent and tissue context influencing conventional outflow as well (Doucette and Walter, 2017; Lütjen-Drecoll and Tamm, 1988; Schachtschabel et al., 2000; Schmidl et al., 2015; Liang et al., 2008; Smid, 2009; Weinreb et al., 2002). Their success established that bioactive lipids can be manipulated safely and effectively in chronic eye disease. Beyond their effects on aqueous humor (AH) dynamics, accumulating evidence suggests that prostanoids also participate in retinal and optic nerve biology, where they may influence neuroinflammatory responses, vascular regulation, and retinal ganglion cell survival (Ward et al., 2026). The major lipid mediator classes implicated in glaucoma, their anatomical localization, proposed mechanisms, biomarker evidence, and therapeutic relevance are summarized in Table 1.

**TABLE 1:** Lipid mediator classes in glaucoma: anatomical localization, biomarker evidence, mechanisms, and therapeutic relevance.

Lipid class	Key mediator(s)/pathway	Main anatomical site(s)	Mechanism in glaucoma	Biomarker evidence: AH/tear/plasma	Therapeutic relevance	Citations
Prostanoids/prostaglandins	PGF2α analogues; latanoprost, travoprost, tafluprost, bimatoprost; latanoprostene bunod	Ciliary muscle, trabecular meshwork, schlemm canal, uveoscleral pathway, conjunctival/uveolymphatic drainage	Increase aqueous outflow through extracellular matrix remodeling, matrix metalloproteinase induction, uveoscleral outflow enhancement, and effects on conventional outflow. NO-donating prostanoid therapy additionally relaxes TM/Schlemm canal	**AH:** prostaglandin-related pathways inferred from lipid mediator studies and drug effects; endogenous prostanoid panels in AH less developed than oxylipins; pharmacodynamic signatures dominate due to widespread topical use. **Tear:** influenced by topical prostaglandin therapy and ocular surface response. **Plasma:** low glaucoma specificity	Most clinically established lipid-based glaucoma therapy. Sustained-release prostanoid implants and dual-mechanism prostanoid/NO donors extend this paradigm	(Doucette and Walter, 2017; Lütjen-Drecoll and Tamm, 1988; Schachtschabel et al., 2000; Schmidl et al., 2015; Mathew and Sivak, 2024), (Uchida et al., 2021; Lima et al., 2012; Gabbs et al., 2015), (Azbukina et al., 2021), (Kawasaki et al., 2002; Patwardhan et al., 2016; Jana et al., 2009; Simón et al., 2019; Sanvicens and Cotter, 2006; Fan et al., 2021; Shiga et al., 2018; Wellcome Trust Case Control Consortium 2 et al., 2014; Chen et al., 2014; Hu et al., 2020; Parton and Del Pozo, 2013)
Oxylipins and oxidized linoleate metabolites	Arachidonic acid; 9-HODE, 13-HODE, 9-KODE, 13-KODE; 12,13-DiHOME	Aqueous humor, trabecular meshwork, ocular surface/tear film, retina	Reflect and may amplify oxidative stress, lipid peroxidation, inflammatory enzyme activity, redox imbalance, and CYP/epoxide hydrolase flux. Oxidized lipids may impair TM homeostasis and contribute to neuroinflammatory signaling	**AH:** targeted lipidomics in POAG identified AA, HODE/KODE species, and 12,13-DiHOME as discriminating inflammatory lipid mediators. **Tear:** some AH oxylipin disturbances appear mirrored in tears. **Plasma:** systemic oxidized lipid patterns may indicate organism-level oxidative susceptibility but lack ocular specificity	Candidate biomarker domain for oxidative/inflammatory glaucoma subtypes. 12,13-DiHOME implicates soluble epoxide hydrolase as a tractable therapeutic target.	(Kolko et al., 2023), (Ward et al., 2026), (Azbukina et al., 2021), (Jager et al., 1993), (Hernández-Corbacho et al., 2017), (Osborne and del Olmo-Aguado, 2013)
Epoxy-fatty acid/soluble epoxide hydrolase pathway	Epoxy-fatty acids; DiHOMEs; soluble epoxide hydrolase	Aqueous humor, vascular endothelium, retina, possibly TM	sEH converts generally protective epoxy-fatty acids into diols. Excess diol formation may promote inflammatory, oxidative, and vascular dysfunction relevant to IOP regulation and neurodegeneration	**AH:** 12,13-DiHOME appears in POAG lipid mediator signatures. **Tear:** possible extension through mirrored oxylipin abnormalities, but validation is limited. **Plasma:** potentially informative for systemic vascular/oxidative state	sEH inhibition is one of the most plausible next-generation lipid therapeutic strategies because it is druggable and acts across inflammatory, vascular, and oxidative pathways	(Ward et al., 2026), (Parton and Del Pozo, 2013; De Ieso et al., 2022; Thorleifsson et al., 2010)
Phospholipids and lysophospholipids	Phosphatidylcholines; lysophosphatidylcholines	Aqueous humor, trabecular meshwork, chamber angle, conjunctiva, plasma	Membrane remodeling and phospholipase activity release bioactive lipid substrates. Altered phospholipid turnover may affect TM mechanotransduction, membrane curvature, receptor localization, inflammation, and outflow resistance	**AH:** altered phosphatidylcholine and lysophosphatidylcholine profiles reported in open-angle and angle-closure glaucoma. **Tear:** affected by ocular surface inflammation and topical medications. **Plasma:** partial overlap with ocular profiles but heavily confounded by systemic metabolism	Useful as biomarker class for membrane remodeling and subtype discrimination. Direct therapeutic targeting is difficult because phospholipid metabolism is broadly required for normal cell function	(Mathew et al., 2026; Yamashita et al., 2017; Rönkkö et al., 2007; Helin et al., 2008; Edwards et al., 2014a)
Autotaxin–lysophosphatidic acid axis	Autotaxin; lysophosphatidylcholine; lysophosphatidic acid; LPA receptors	Aqueous humor, trabecular meshwork, schlemm canal, lamina cribrosa	ATX converts lysophosphatidylcholine to LPA. LPA signaling promotes actomyosin contractility, stress-fiber formation, extracellular matrix deposition, connective tissue growth factor activity, Rho/ROCK signaling, and reduced aqueous outflow. May also contribute to lamina cribrosa fibrosis	**AH:** increased ATX/LPA pathway activity in glaucoma, especially secondary open-angle and exfoliation glaucoma; associated with IOP and trabeculectomy outcomes. **Tear:** not yet a mature biomarker domain. **Plasma:** likely less specific than AH.	Highly translational target for IOP lowering and antifibrotic therapy. ATX inhibitors and LPA-axis modulation may complement ROCK inhibitors or surgery-related antifibrotic strategies	(Livne-Bar et al., 2017), (Igarashi et al., 2020), (Cabrerizo et al., 2017; Honjo et al., 2018; Igarashi et al., 2018; Iyer et al., 2012; Ho et al., 2020)
PAF-related lipids	Platelet-activating factor; lyso-PAF	Aqueous humor, ocular inflammatory tissues, vascular/neural compartments	Links phospholipid remodeling to inflammation, vascular permeability, platelet activation, excitatory neurotransmission, and possibly ocular hypertension	**AH:** elevated lyso-PAF identified in glaucoma lipidomics. **Tear:** related disturbances may be detectable but need validation. **Plasma:** nonspecific because PAF biology is systemic and inflammatory	Older preclinical work supports PAF antagonism in ocular hypertension models, but modern translation remains underdeveloped. May be most useful for inflammatory or vascular glaucoma phenotypes	(Milbeck and Bhattacharya, 2020; Rosenbaum et al., 1991; Bazan and Allan, 1997; Jager et al., 1993)
Sphingolipids	Sphingomyelins; ceramides; sphingosine; sphingosine-1-phosphate	Aqueous humor, trabecular meshwork, retina, optic nerve, possibly lamina cribrosa	Ceramides promote apoptosis, oxidative stress, mitochondrial dysfunction, ER stress, inflammatory amplification, and possibly TM cell dysfunction. S1P may promote survival, migration, fibrosis, or inflammation depending on receptor context	**AH:** altered sphingolipid and ceramide profiles reported in glaucoma; sphingomyelins and ceramides appear prominent in angle-closure disease and pseudoexfoliation glaucoma. **Tear:** less established. **Plasma:** systemic confounding substantial	Potential biomarker class for inflammatory, fibrotic, or neurodegenerative phenotypes. Therapeutic modulation of the sphingolipid rheostat is attractive but challenging due to pathway pleiotropy	(Buisset et al., 2019), (Chistyakov et al., 2020; Simon et al., 2021; Mondal and Mandal, 2019; Spiegel and Milstien, 2003), (Mathew et al., 2021; Brust et al., 2008; Sakai et al., 2009; Maihöfner et al., 2001; Yasuda et al., 2016)
Endocannabinoids and cannabinoid-sensitive lipid signaling	Anandamide; 2-arachidonoylglycerol; CB1, CB2, TRPV1, GPR18 pathways; FAAH/MAGL-regulated tone	Trabecular meshwork, ciliary epithelium, retina, müller glia, retinal ganglion cells, ocular vasculature	Modulates IOP, aqueous outflow, ciliary secretion, vascular tone, glial inflammation, calcium signaling, excitotoxicity, and RGC survival. TRPV1 links pressure stress to calcium-dependent RGC injury, while cannabinoid pathways may counter inflammation and neurodegeneration	**AH:** direct clinical lipidomic evidence less mature than for oxylipins or ATX/LPA. **Tear:** potentially accessible but medication and surface confounding are major issues. **Plasma:** poor ocular specificity	Strong therapeutic potential through topical receptor-selective agonists, FAAH/MAGL inhibition, GPR18/TRPV1 modulation, or sustained local delivery. Translation requires avoiding systemic psychoactive and cardiovascular effects	(Aribindi et al., 2013; Yazulla, 2008; Cairns et al., 2016; Cécyre et al., 2013; Gagliano et al., 2011; Rapino et al., 2018; Warjri et al., 2025; Jay and Green, 1983; Plange et al., 2007; Passani et al., 2020; Lindner et al., 2023; Bouskila et al., 2013; Krishnan and Chatterjee, 2012; Zhong et al., 2005; Njie et al., 2008a), (Tie et al., 2020; Vranka et al., 2018; Acott and Kelley, 2008; Stamer and Acott, 2012; Morgan et al., 2015; Izzotti et al., 2006)
Specialized pro-resolving mediators, including DHA-derived neuroprotective lipids	Lipoxin A4, lipoxin B4, resolvins, protectins/neuroprotectin D1, maresins	Retina, optic nerve head astrocytes, microglia, müller glia, retinal ganglion cells, optic nerve injury sites	Promote active resolution of inflammation, restrain astrocyte and microglial activation, reduce inflammatory chemokine signaling, counter oxidative and inflammatory stress, and support retinal ganglion cell survival after acute or chronic injury. DHA-derived mediators such as neuroprotectin D1 may be especially relevant to RGC resilience after optic nerve injury	**AH:** Limited direct clinical biomarker evidence. **Tear**: Plausible future marker of failed inflammatory resolution but not yet validated. **Plasma**: Systemic resolution tone may be measurable but nonspecific and confounded by diet/systemic inflammation. Retina/optic nerve: Strongest mechanistic evidence from experimental injury and glaucoma models	One of the most compelling pressure-independent therapeutic strategies. Lipoxin, resolvin, protectin/NPD1, or maresin analogues could target neuroinflammation, oxidative stress, and RGC vulnerability in patients progressing despite IOP control. Key challenges include mediator stability, ocular delivery, receptor specificity, dose timing, and therapeutic window	(Mathew and Sivak, 2024), (Zhou et al., 2026), (Qin et al., 2008), (Azbukina et al., 2021), (Nucci et al., 2007; Frank et al., 2022; Livne-Bar et al., 2024; Kumar et al., 2026; Maurya et al., 2024)
Sterol and neutral lipid remodeling	Cholesterol esters; triglycerides; sterol-related lipid changes	Aqueous humor, plasma, pseudoexfoliation-associated anterior segment tissues	May reflect altered lipid transport, membrane turnover, extracellular material deposition, and metabolic stress, especially in pseudoexfoliation glaucoma	**AH:** cholesterol esters, phosphatidylcholines, triglycerides, and ceramides altered in pseudoexfoliation glaucoma **Tear:** not yet well established. **Plasma:** Systemic lipid metabolism strongly confounds interpretation	More useful for subtype characterization than immediate drug targeting. May help distinguish pseudoexfoliation-related lipid remodeling from POAG or angle-closure signatures	(Tanaka et al., 2020), (Tiberi and Chiurchiù, 2021), (Järvinen et al., 2002; Green, 1979; Nucci et al., 2008)

Integrated lipidomic signatures are composite pathway-level measures, such as oxylipin balance, epoxy-to-diol ratio, ceramide/sphingomyelin balance, and pro-inflammatory/pro-resolving mediator balance. They may better capture glaucoma biology than single analytes, with interpretation depending on sampling compartment.

Prostanoids are an arachidonic acid–derived oxylipin subclass.

Targeted lipidomic studies in human AH have implicated arachidonic acid (AA); oxidized linoleate products including 9-hydroxyoctadecadienoic acid (9-HODE) and 13-hydroxyoctadecadienoic acid (13-HODE); their corresponding keto derivatives, 9-oxo-octadecadienoic acid (9-KODE) and 13-oxo-octadecadienoic acid (13-KODE); and 12,13-dihydroxyoctadecenoic acid (12,13-DiHOME), a diol formed downstream of linoleic acid epoxides by epoxide hydrolases (Umeno et al., 2019; Azbukina et al., 2021).

A lipid panel measured during surgery represents an integrated signal from mechanical stress, topical therapy, ocular surface irritation, and systemic metabolic status, rather than a single disease-specific event. This point is highlighted by Mathew and colleagues, who profiled COX- and LOX-derived lipid mediators in AH and found a focused increase in arachidonic acid and LXA4 in glaucomatous eyes (Mathew and Sivak, 2024; Mathew et al., 2026). This issue is particularly relevant to prostaglandin analogue therapy, as subsequent experimental evidence has demonstrated that latanoprost can directly induce an AA/LXA4 lipid-signaling circuit in trabecular meshwork cells (Mathew et al., 2026). The implications of treatment exposure for lipidomic interpretation are discussed in detail in Section 6.1.

### 2.2 Phospholipids, lysophospholipids, and PAF-related lipids

Phospholipids are the primary structural lipids of cell membranes, but their hydrolysis generates several bioactive products. Phospholipase A2-mediated cleavage of membrane phospholipids releases free fatty acids such as AA and forms lysophospholipids, which themselves act as signaling molecules or precursors (Yamashita et al., 2017; Rönkkö et al., 2007; Helin et al., 2008). In glaucoma, phospholipase activity has been detected in relevant anterior segment tissues, including chamber angle and conjunctiva, supporting the idea that local phospholipid remodeling participates in disease-related signaling (Rönkkö et al., 2007; Helin et al., 2008).

Lysophospholipids couple mechanical or inflammatory membrane stress to downstream signaling cascades. In AH studies, changes in phosphatidylcholines (PCs) and lysophosphatidylcholines (LPCs) have been reported in open-angle and angle-closure glaucoma (Edwards et al., 2014a; Cabrerizo et al., 2017). These shifts reflect altered membrane turnover, oxidative remodeling, or inflammatory activation within tissues responsible for AH homeostasis.

### 2.3 The autotaxin–LPA axis

Among lysophospholipid pathways, the autotaxin–lysophosphatidic acid (ATX–LPA) axis has emerged as one of the most translationally active areas in glaucoma lipid research. Autotaxin is a secreted lysophospholipase D that converts lysophosphatidylcholine into lysophosphatidic acid, a bioactive lipid that signals through G-protein-coupled LPA receptors. Human AH studies found increased autotaxin/LPA pathway activity in glaucoma, particularly in secondary open-angle and exfoliation glaucoma, with significant associations between ATX–LPA measures and IOP (Igarashi et al., 2020; Honjo et al., 2018). Related work also found that aqueous autotaxin and LPA levels predict trabeculectomy outcomes and fibrotic bleb remodeling, further linking this pathway to postoperative wound healing and pressure control (Igarashi et al., 2018). In Posner–Schlossman syndrome, aqueous autotaxin was elevated, correlated with IOP, and was experimentally linked to fibrogenic responses in trabecular meshwork cells, especially in the context of cytomegalovirus infection (Igarashi et al., 2020).

Mechanistically, the ATX–LPA axis offers a plausible link between lipid metabolism and increased outflow resistance. In preclinical trabecular meshwork and Schlemm canal–related models, LPA receptor signaling promotes actomyosin contractility, stress-fiber formation, extracellular matrix production, and reduced aqueous outflow, effects that converge in part on Rho/Rho kinase signaling. Preclinical work identified this pathway as a molecular target for lowering IOP and showed that pharmacologic ATX inhibition, including with PF-8380, reduced IOP in experimental models (Iyer et al., 2012). Subsequent human aqueous-humor and cellular studies reported increased autotaxin, lysophosphatidylcholine, LPA, and connective tissue growth factor in open-angle glaucoma, while inflammatory and profibrotic stimuli increased autotaxin expression in human trabecular meshwork cells; these responses were attenuated by pathway inhibition, supporting an ATX–LPA–associated profibrotic mechanism (Ho et al., 2020).

Lamina cribrosa fibrosis is a central component of glaucomatous optic nerve head remodeling, and O’Regan, O’Brien, and colleagues have argued that the LPA axis contributes to fibrotic remodeling in both trabecular meshwork and lamina cribrosa tissues (O’Regan et al., 2021). However, lamina cribrosa-specific evidence remains less mature than the anterior-segment literature. Human optic nerve data are also not fully concordant with AH findings: Milbeck and Bhattacharya reported altered lysophospholipid profiles and pathway enzyme changes in glaucomatous optic nerve tissue, highlighting that LPA-related signaling varies by compartment, disease stage, or tissue response (Milbeck and Bhattacharya, 2020).

### 2.4 Platelet activating factor-related lipids

Platelet activating factor **(**PAF) is a potent phospholipid-derived mediator involved in inflammation, platelet activation, vascular permeability, and excitatory neurotransmission (Rosenbaum et al., 1991; Bazan and Allan, 1997; Jager et al., 1993). In the eye, PAF and related ether phospholipids are relevant because they link inflammatory signaling with vascular and neural responses that may influence both anterior and posterior segment pathology. Ocular tissues can synthesize PAF under inflammatory conditions, and experimental work showed that PAF antagonism could prevent prostaglandin-induced ocular hypertension in rabbits, suggesting that PAF signaling may participate in pressure-related inflammatory responses rather than acting solely as a downstream bystander (Rosenbaum et al., 1991; Jager et al., 1993). More recent glaucoma lipidomics studies have identified elevated lyso-PAF in aqueous humor, renewing interest in whether PAF-related lipid remodeling contributes to altered aqueous-humor composition, trabecular meshwork stress, or broader inflammatory signaling in glaucoma (Azbukina et al., 2021; Chistyakov et al., 2020).

However, compared with prostaglandins, endocannabinoids, and the autotaxin–LPA pathway, the PAF axis remains less well developed translationally. Existing evidence is still largely indirect, deriving from ocular inflammation models, pharmacologic antagonist studies, and lipidomic correlations rather than from human interventional studies. Nevertheless, PAF-related biology may prove important because it sits at the crossroads of inflammation, vascular regulation, blood–ocular barrier permeability, and neuronal stress. These features make PAF-related lipids plausible contributors to glaucoma-relevant tissue responses, particularly in settings where inflammatory or vascular dysregulation overlaps with IOP-related stress. Further work will be needed to determine whether elevated lyso-PAF reflects a causal mediator, a compensatory remodeling product, or a biomarker of inflammatory lipid metabolism in glaucomatous eyes.

### 2.5 Sphingolipids

Sphingolipids have emerged as another major lipid class relevant to glaucoma. Sphingolipids include sphingomyelins (SMs), ceramides (Cers), sphingosine, and sphingosine-1-phosphate (S1P) (Simon et al., 2021; Mondal and Mandal, 2019). Ceramides are often associated with apoptosis, oxidative stress, and inflammatory amplification, while S1P can promote survival, migration, fibrosis, or inflammation depending on receptor context and tissue environment. The balance between these pathways is sometimes described as the “sphingolipid rheostat”. (Spiegel and Milstien, 2003).

In ocular studies, altered sphingolipid profiles have been reported in human glaucomatous AH and TM, as well as in murine models (Aljohani et al., 2013; Edwards et al., 2014a; Edwards et al., 2014b; Aribindi et al., 2013). These findings are notable because they place sphingolipid disruption directly in the outflow apparatus, not merely in injured neural tissues. Ceramide accumulation could influence membrane microdomains, mechanotransduction, cell survival, extracellular matrix production, and cytokine responses in TM cells. In posterior segment tissues, ceramides facilitate mitochondrial dysfunction, endoplasmic reticulum stress, and pro-apoptotic signaling in neurons and glia (Simon et al., 2021; Mondal and Mandal, 2019). Thus, sphingolipids are well positioned to connect anterior segment dysfunction with posterior segment neurodegeneration.

### 2.6 Endocannabinoids

The endocannabinoid system includes endogenous ligands such as N-arachidonoylethanolamine (anandamide; AEA) and 2-arachidonoylglycerol (2-AG), together with their biosynthetic and degradative enzymes and receptors including cannabinoid receptor 1 (CB1), cannabinoid receptor 2 (CB2), transient receptor potential vanilloid 1 (TRPV1), and G-protein-coupled receptor 18 (GPR18) (Yazulla, 2008; Cairns et al., 2016; Cécyre et al., 2013). This system is highly relevant to glaucoma because it operates in both the anterior segment and retina. Endocannabinoids can lower IOP, modulate outflow facility, influence ciliary epithelium secretion, alter vascular tone, suppress glial inflammation, and reduce excitotoxic or pressure-induced neuronal injury (Gagliano et al., 2011; Rapino et al., 2018; Warjri et al., 2025; Jay and Green, 1983; Plange et al., 2007; Passani et al., 2020; Lindner et al., 2023).

Experimental work has demonstrated cannabinoid receptor expression in TM cells, retinal neurons, and Müller glia (Cécyre et al., 2013; Bouskila et al., 2013; Krishnan and Chatterjee, 2012; Zhong et al., 2005). CB2 receptor signaling, for example, can enhance outflow facility in TM-related systems, while AEA and 2-AG analogues have been shown to influence aqueous outflow (Njie et al., 2008a; Njie et al., 2008b). In the retina, cannabinoid signaling can reduce inflammatory activation and protect RGCs in pressure or ischemia-related injury models (Krishnan and Chatterjee, 2012; Nucci et al., 2007).

### 2.7 Specialized pro-resolving mediators

Specialized pro-resolving mediators (SPMs) are enzymatically generated lipid mediators that actively terminate inflammation, restore tissue homeostasis, and promote repair rather than simply suppressing immune activity. The main families include arachidonic acid (AA)-derived lipoxins (Mathew and Sivak, 2024), eicosapentaenoic acid (EPA)-derived E-series resolvins; and docosahexaenoic acid (DHA)-derived D-series resolvins, protectins, including neuroprotectin D1 (NPD1), and maresins. Rather than blocking inflammation broadly, SPM-based strategies aim to restore endogenous resolution circuits that become insufficient during chronic ocular stress.

The strongest glaucoma-relevant evidence comes from the lipoxin pathway. In a landmark study, Livne-Bar et al. (2017) found that lipoxin A4 (LXA4) and lipoxin B4 (LXB4) protect RGCs in models of acute and chronic injury (Livne-Bar et al., 2017; Frank et al., 2022). In experimental glaucoma, therapeutic LXB_4_ preserved RGC function and survival, supporting an astrocyte-derived lipid circuit that restrains neurodegeneration rather than amplifying it. Subsequent work has strengthened this model and revealed additional lipoxin effects on retinal inflammation. Livne-Bar et al. (2024) showed that LXA_4_ and LXB_4_ reduce acute inner retinal inflammation through CXCR3 signalling, and that CXCR3 inhibition reduced neuroinflammation due to LPS (Livne-Bar et al., 2024). Additionally, Gronert, Flanagan and colleagues have studied potent LXB_4_ activities in microglia, astrocytes, and Müller glia (Kumar et al., 2026; Maurya et al., 2024; Karnam et al., 2024).

DHA-derived protectins provide a second relevant SPM axis. Qin et al. (2008) showed that neuroprotectin D1 (NPD1) delayed RGC death after optic nerve transection, increasing RGC survival (30% at 2 weeks) compared with untreated injured controls (12% at 2 weeks), indicating that DHA-derived resolution mediators can directly support neuronal survival after retinal injury (Qin et al., 2008). Recent non-ocular receptor studies have identified GPR37 as a receptor for NPD1/protectin D1 in neuroimmune and pain-signaling contexts, providing a potential receptor framework that could inform future ocular studies (Xu et al., 2025). However, extrapolation to glaucoma should remain cautious, because direct validation of GPR37-mediated NPD1/protectin D1 signaling in glaucomatous retinal ganglion cell injury is currently limited.

An important mechanistic link that warrants greater consideration is the crosstalk between lipid metabolism, mitochondrial dysfunction, and RGC death. Mitochondria are not only major sites of energy metabolism but also critical regulators of apoptosis, and alterations in mitochondrial membrane lipids can directly influence this process. In particular, cardiolipin, a mitochondria-specific phospholipid, is essential for maintaining respiratory-chain organization and mitochondrial membrane integrity; its oxidation promotes cytochrome c dissociation and facilitates mitochondrial outer-membrane permeabilization, thereby linking lipid peroxidation to intrinsic apoptosis (Kagan et al., 2009; Iverson and Orrenius, 2004; Houtkooper and Vaz, 2008). Similarly, bioactive lipid metabolites such as ceramide can act directly at mitochondria to promote mitochondrial dysfunction, membrane permeabilization, and downstream caspase activation (Fisher-Wellman et al., 2021; Hernández-Corbacho et al., 2017). This relationship may be particularly relevant to RGCs, which have exceptionally high energetic demands and are therefore highly dependent on mitochondrial integrity (Osborne and del Olmo-Aguado, 2013). Beyond apoptosis, lipid dysregulation may also promote ferroptosis, an iron-dependent form of regulated cell death characterized by the accumulation of toxic lipid peroxides and failure of antioxidant defenses. Experimental glaucoma and optic nerve injury models have demonstrated increased iron and lipid peroxidation together with reduced glutathione peroxidase 4 (GPX4), while pharmacological inhibition of ferroptosis preserves RGC survival and mitochondrial function (Guo et al., 2022; Yao et al., 2023). Importantly, cholesterol homeostasis represents another lipid-dependent determinant of RGC viability. ABCA1-mediated cholesterol efflux is critical for maintaining retinal cholesterol balance, and impaired ABCA1 function produces retinal cholesterol accumulation, altered oxidative phosphorylation, impaired autophagy, and progressive RGC loss (Yang et al., 2023). More recent evidence further suggests that restoration of AIBP–ABCA1-mediated cholesterol homeostasis can reduce cholesterol accumulation, mitochondrial dysfunction, and neuroinflammation while protecting RGCs in experimental glaucoma (Ju et al., 2025). Collectively, these findings suggest that altered lipid metabolites may not simply represent biomarkers of glaucomatous disease, but could participate directly in mitochondrial dysfunction, apoptosis, ferroptosis, and disruption of cholesterol homeostasis, providing a biologically plausible pathway through which systemic or retinal lipid abnormalities could contribute to RGC vulnerability.

## 3 Mechanistic roles of lipid mediators in glaucoma pathogenesis

### 3.1 Lipid signaling and IOP regulation

AH formation and drainage depend on a coordinated balance between ciliary body secretion, conventional outflow through the TM/Schlemm canal pathway, and uveoscleral outflow (Umeno et al., 2019). Lipid mediators influence all three domains.

Within the outflow apparatus, lipid mediators alter cytoskeletal tone, extracellular matrix remodeling, cellular adhesiveness, permeability, and inflammatory state. TM cells sense and respond to pressure, matrix stiffness, oxidative injury, and cytokines (Tie et al., 2020; Vranka et al., 2018; Acott and Kelley, 2008; Stamer and Acott, 2012; Morgan et al., 2015). Lipid mediators provide an efficient biochemical language for this response because mechanical or oxidative perturbation of membranes rapidly releases signaling lipids through phospholipase activation and oxidation. The resulting mediators feedback on actomyosin contractility, matrix metalloproteinases, fibrotic signaling, and endothelial behaviour in Schlemm canal.

In glaucomatous tissue, changes in phospholipids, ceramides, and related mediators reduce the capacity of the outflow pathway to maintain homeostasis under chronic stress. This concept is particularly important translationally because it reveals that lipid abnormalities detected in AH are not just downstream markers of disease severity but represent contributing factors to pressure dysregulation itself.

### 3.2 Trabecular meshwork dysfunction and extracellular matrix remodeling

TM dysfunction is a central feature of POAG, and lipid biology is intertwined with this process. Elevated substrate stiffness alters the transcriptome and proteome of human TM cells (Tie et al., 2020; Vranka et al., 2018; Morgan et al., 2015). Because membrane lipid composition influences mechanosensitive signaling, focal adhesion behavior, caveolar dynamics, and receptor clustering, stiffness-related changes in TM cells are not purely protein driven. Phospholipid remodeling alters cell membrane curvature and receptor localization, while sphingolipid-rich microdomains can influence signal transduction and apoptosis. Ceramide accumulation, in particular, reinforces maladaptive stress responses.

Extracellular matrix turnover is also influenced by lipid mediators. Prostaglandins are known to affect matrix metalloproteinase expression, contributing to increased uveoscleral outflow (Schachtschabel et al., 2000; Schmidl et al., 2015; Weinreb et al., 2002). Similar matrix-directed effects arise from other oxylipins or sphingolipid pathways. Oxidized phospholipids and lysophospholipids can activate inflammatory or fibrotic signaling cascades that promote matrix deposition and reduced outflow. The result can be a self-reinforcing loop in which mechanical stress alters lipid signaling, which stiffens the tissue further, generating even more abnormal lipid responses.

### 3.3 Oxidative stress and lipid peroxidation

The eye is particularly susceptible to oxidative stress because of its high oxygen exposure, abundance of polyunsaturated lipids, light exposure, and metabolically active neural tissues (Izzotti et al., 2006; He et al., 2017). In glaucoma, oxidative stress has long been implicated in TM dysfunction, mitochondrial injury, and optic nerve degeneration (Izzotti et al., 2006). Lipid peroxidation is one of the main ways in which oxidative stress becomes biologically consequential.

Oxidized lipids serve as biomarkers of oxidative burden and propagate damage by activating inflammatory receptors, modulating ion channels, and inducing mitochondrial dysfunction. The recurring identification of HODE/KODE-related species in glaucoma studies suggests that oxidized linoleate metabolism functions as an accessible window into tissue redox status (Umeno et al., 2019; Azbukina et al., 2021; Chistyakov et al., 2020). Similarly, CYP1B1 biology links lipid metabolism to oxidative homeostasis and inherited glaucoma susceptibility (Vasiliou and Gonzalez, 2008; Falero-Perez et al., 2018). This reinforces the idea that oxidative stress in glaucoma should not be conceptualized only in generic reactive oxygen species terms; it is also a disease of oxidized lipid signaling.

### 3.4 Neuroinflammation and glial activation

Glial activation has become a key theme in contemporary glaucoma research. Microglia, astrocytes, and Müller glia respond to elevated pressure, ischemia, mitochondrial distress, and neuronal injury with changes in cytokine release, complement activation, phagocytic behavior, and metabolic state (Mathew et al., 2021). Lipid mediators are deeply woven into these responses. COX-2 induction after retinal injury can amplify prostaglandin signaling and inflammatory gene expression (Brust et al., 2008; Sakai et al., 2009; Maihöfner et al., 2001; Yasuda et al., 2016). Phospholipase activation supplies AA substrate for eicosanoid production (Rönkkö et al., 2007; Helin et al., 2008). Ceramide accumulation can activate stress kinases and inflammatory programs (Simon et al., 2021; Mondal and Mandal, 2019). PAF-related signaling promotes excitatory neurotransmission and vascular-inflammatory coupling (Rosenbaum et al., 1991; Bazan and Allan, 1997; Jager et al., 1993). By contrast, endocannabinoid/cannabinoid and specialized pro-resolving mediator pathways restrain glial activation and promote a return toward pro-resolving states (Tanaka et al., 2020; Tiberi and Chiurchiù, 2021). Lipoxins have been shown to suppress retinal neuroinflammation and inhibit astrocyte, microglial, and Müller glial activation, supporting endogenous mechanisms of inflammatory resolution and neuroprotection in the retina (Livne-Bar et al., 2017; Livne-Bar et al., 2024).

Chronic glaucoma involves persistent low-level activation that fails to resolve, not continuous high-intensity inflammation. In that setting, blocking a single inflammatory pathway is unlikely to be sufficient; enhancing resolution through lipoxins or related mediators may restore glial homeostasis more effectively than broad suppression.

### 3.5 Excitotoxicity, calcium dysregulation, and neuronal death

Excitotoxicity has long been proposed as a contributor to glaucomatous neurodegeneration, particularly because RGCs are vulnerable to sustained disturbances in glutamatergic signaling and intracellular calcium homeostasis. Excessive glutamate receptor activation can permit pathologic calcium influx, activating calcium-dependent proteases, phospholipases, mitochondrial permeability transition, oxidative stress, and downstream apoptotic pathways (Casson, 2006).

Several lipid mediators intersect with this pathway more directly than simple inflammatory signaling. Sappington et al. demonstrated that elevated hydrostatic pressure increased intracellular calcium in RGCs and induced apoptosis substantially through TRPV1, a calcium-permeable channel linking pressure-related stress to neuronal death (Sappington et al., 2009). TRPV1 antagonism reduced pressure-induced RGC apoptosis, while extracellular calcium chelation reduced apoptosis under elevated pressure conditions, supporting a calcium-dependent mechanism (Sappington et al., 2009). This provides a mechanistic bridge between intraocular pressure-related stress and lipid-sensitive calcium signaling, particularly because TRPV1 activity can be modulated by endogenous lipid ligands, including cannabinoid-related molecules and arachidonic acid-derived metabolites (Mathew and Sivak, 2024; Yazulla, 2008; Järvinen et al., 2002; Green, 1979; Nucci et al., 2008).

AA also has a complex relationship with excitotoxic injury. Kawasaki et al. showed that exposure of cultured rat RGCs to glutamate produced substantial RGC loss, while low concentrations of AA significantly attenuated glutamate-induced neurotoxicity (Kawasaki et al., 2002). Mechanistically, AA reduced glutamate-, AMPA-, and kainate-induced intracellular calcium accumulation and inhibited non-NMDA ionotropic receptor currents in a dose-dependent manner (Kawasaki et al., 2002). However, this effect was concentration-dependent, as AA became directly toxic to RGCs at higher concentrations (Kawasaki et al., 2002). AA can either buffer excitotoxic calcium entry or contribute to neuronal injury depending on concentration, metabolic fate, receptor context, and local oxidative environment.

Ceramides further amplify neuronal vulnerability by promoting endoplasmic reticulum stress, disrupting mitochondrial function, increasing mitochondrial membrane permeability, and facilitating apoptotic signaling, as supported by broader neurodegeneration and mitochondrial apoptosis literature (Patwardhan et al., 2016; Jana et al., 2009). In retinal models, ceramide accumulation has been linked to oxidative stress-induced neuronal apoptosis, mitochondrial pathway activation, and increases in cytosolic and mitochondrial Ca^2+^, although much of this evidence derives from photoreceptor or mixed retinal neuronal systems rather than glaucoma-specific RGC models (Simón et al., 2019; Sanvicens and Cotter, 2006). More directly, Fan et al. reported ceramide-induced cytotoxicity in retinal ganglion cell models and showed that intravitreal C2-ceramide induces apoptosis in the RGC layer with associated glial activation (Fan et al., 2021). Thus, while direct evidence linking ceramide accumulation to excitotoxic RGC death in glaucoma remains limited, sphingolipid-mediated mitochondrial dysfunction provides a mechanism by which inflammatory lipid remodeling converges with neuronal death pathways.

### 3.6 Genetic evidence for lipid pathway involvement

Genetic evidence further supports the involvement of lipid-related pathways in glaucoma susceptibility. ABCA1, which encodes an ATP-binding cassette transporter responsible for cholesterol and phospholipid efflux to apolipoprotein A-I, is among the most consistently replicated POAG susceptibility loci. ABCA1 is expressed in the trabecular meshwork, Schlemm canal endothelium, retina, retinal ganglion cell layer, and optic nerve. Functional studies indicate that reduced ABCA1 activity promotes retinal cholesterol accumulation, mitochondrial dysfunction, impaired autophagy, neuroinflammation, and retinal ganglion cell loss. In the trabecular meshwork, ABCA1 also influences aqueous outflow through caveolin-1/endothelial nitric oxide synthase signalling, providing a mechanistic link between cholesterol homeostasis and intraocular pressure regulation (Yang et al., 2023; Shiga et al., 2018; Wellcome Trust Case Control Consortium 2 et al., 2014; Chen et al., 2014; Hu et al., 2020). Together, these genetic and mechanistic data indicate that altered lipid efflux is not simply reflected in glaucomatous tissue but may contribute to dysregulated aqueous outflow and RGC vulnerability, giving the lipid-metabolism hypothesis a causal genetic anchor that complements the biomarker evidence discussed in Section 4.

The CAV1/CAV2 locus represents another important connection between glaucoma genetics and lipid biology. Caveolin-1 and caveolin-2 are structural components of caveolae, cholesterol- and sphingolipid-rich membrane microdomains that organize mechanotransduction, lipid trafficking, endothelial nitric oxide synthase activity, and receptor signalling (Parton and Del Pozo, 2013; De Ieso et al., 2022). Variants at the CAV1/CAV2 locus have been associated with POAG and intraocular pressure in multiple populations (Thorleifsson et al., 2010; Loomis et al., 2014; Kim et al., 2015). In ocular outflow tissues, altered caveolin signalling may affect trabecular meshwork and Schlemm canal mechanosensing, cytoskeletal organization, nitric oxide bioavailability, and aqueous outflow resistance (De Ieso et al., 2022; Elliott et al., 2016).

More recently, an integrative multi-omics study identified PLCE1 and ALAS2 as candidate genes linking lipid metabolism-derived protein acylation with POAG (Zhao et al., 2025). PLCE1 encodes phospholipase C epsilon 1, an enzyme that hydrolyses phosphatidylinositol 4,5-bisphosphate to generate diacylglycerol and inositol trisphosphate, thereby activating protein kinase C and intracellular calcium signalling (Cocco et al., 2015). In the multi-omics analysis, myristoylated PLCE1 was proposed to promote trabecular meshwork fibrosis through interactions involving collagen type IV alpha 3 (Zhao et al., 2025). However, this proposed mechanism was derived predominantly from integrated transcriptomic, bioinformatic, machine-learning, and single-cell analyses and has not yet been established through glaucoma-specific experimental manipulation of PLCE1.

The same study identified ALAS2, which encodes the erythroid-specific rate-limiting enzyme in heme biosynthesis, as another candidate gene associated with lipid-derived acylation signatures in POAG (Zhao et al., 2025). The authors proposed that ALAS2 contributes to the availability of acyl-CoA substrates involved in protein acylation. Nevertheless, ALAS2 is not an established glaucoma susceptibility locus or a conventional lipid-metabolism gene, and its proposed role in POAG currently requires independent experimental validation. Collectively, these findings suggest that glaucoma-associated genetic and molecular variation may influence cholesterol efflux, caveolar organization, phosphoinositide signalling, nitric oxide regulation, protein acylation, and aqueous outflow. However, the evidence is strongest for ABCA1 and CAV1/CAV2, whereas PLCE1 and ALAS2 should presently be regarded as emerging multi-omics candidates.

These genetic findings complement the broader lipid-signalling mechanisms described throughout this section. As summarized in Figure 2, glaucoma-associated genetic and molecular alterations may disrupt interconnected pathways involving cholesterol efflux and caveolar organization, phospholipid and sphingolipid metabolism, oxylipin production, endocannabinoid signalling, and specialized pro-resolving mediators. These alterations may converge on impaired aqueous outflow, trabecular meshwork fibrosis, oxidative and mitochondrial stress, neuroinflammation, and retinal ganglion cell degeneration. Figure 2 also distinguishes comparatively well-supported mechanisms, such as ABCA1-and CAV1/CAV2-associated regulation of cholesterol homeostasis and aqueous outflow, from emerging pathways involving PLCE1-and ALAS2-associated protein acylation.

**FIGURE 2:**
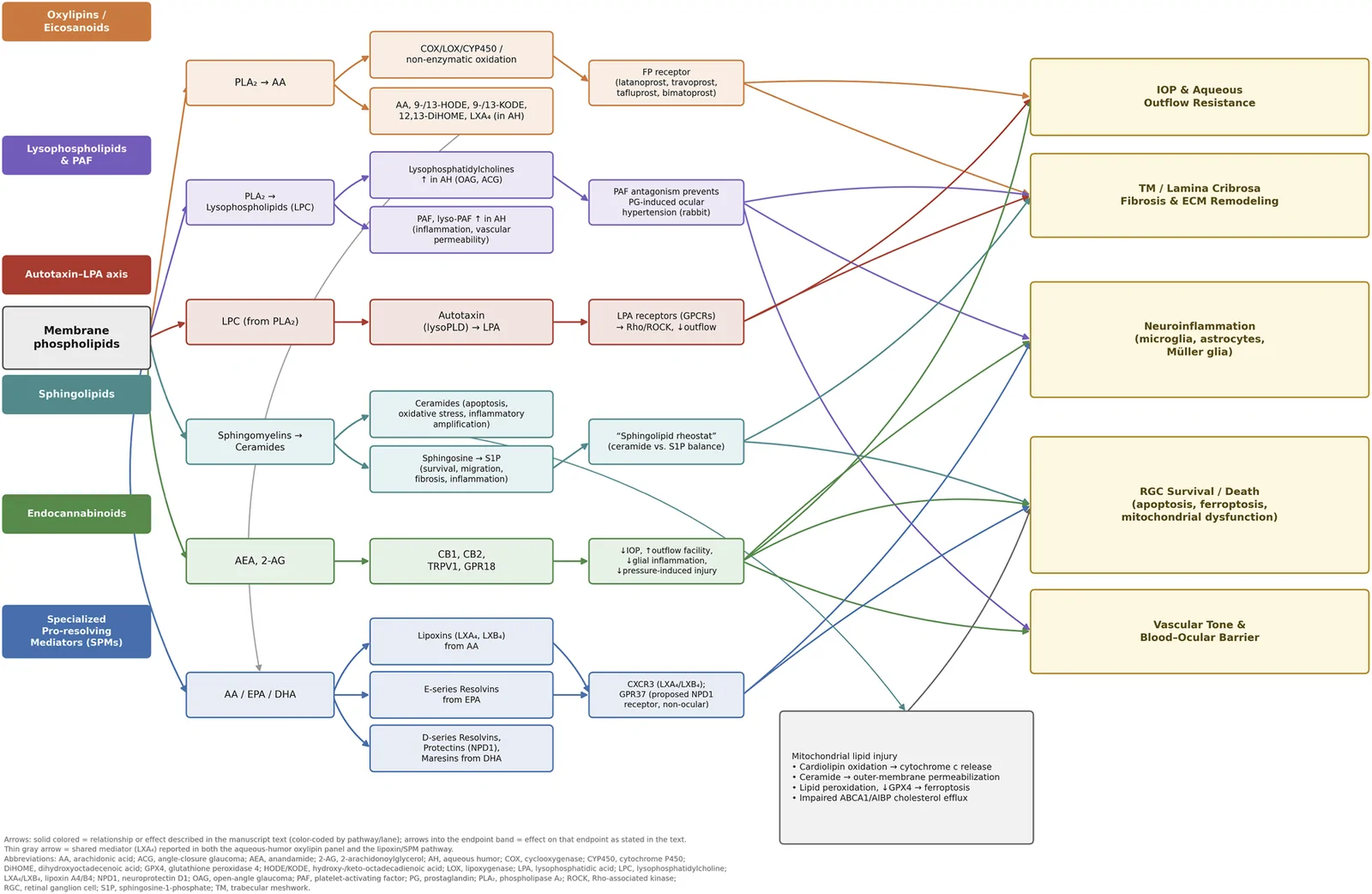
Lipid signaling pathways and glaucoma-associated genes implicated in glaucoma. Major bioactive lipid pathways include oxylipins/eicosanoids, lysophospholipids, the autotaxin–LPA axis, sphingolipids, endocannabinoids, and specialized pro-resolving mediators, with proposed effects on intraocular pressure and aqueous outflow, fibrosis and extracellular matrix remodeling, neuroinflammation, retinal ganglion cell survival, vascular function, and mitochondrial injury. Glaucoma-associated genes implicated in these pathways include ABCA1, CAV1/CAV2, PLCE1, and ALAS2, which have been linked to cholesterol efflux and homeostasis, caveolar organization and aqueous outflow, phospholipid signaling and protein acylation, and heme/mitochondrial metabolism, respectively. Colored arrows indicate pathway-specific relationships, while gray arrows indicate shared metabolic connections. Solid and dashed pathway relationships distinguish comparatively well-supported mechanisms from emerging or proposed associations.

## 4 Evidence for lipidomic biomarkers in glaucoma

### 4.1 Aqueous humor

AH has been the most informative clinical substrate for ocular lipidomics because it is anatomically proximate to the tissues responsible for IOP regulation and is accessible during cataract or glaucoma surgery. Early studies demonstrated altered phospholipid and sphingolipid composition in glaucomatous AH, including increased ceramide-related species (Aljohani et al., 2013; Edwards et al., 2014a).

Orthogonal aqueous-humor biomarker evidence also supports disease-specific molecular heterogeneity. Proteomic profiles differ between early and advanced POAG, while a systematic review of angle-closure glaucoma identified elevations in inflammatory, angiogenic, and extracellular-matrix-associated mediators, including VEGF, IL-8, MMP-2, MCP-1, and TIMP-1 (Zhou et al., 2026; Huang et al., 2026). Cabrerizo and colleagues profiled 110 AH lipid species in 10 open-angle glaucoma (OAG) patients versus 10 controls and found 37 species significantly altered; the majority were diacylglycerophosphocholines and 1-ether, 2-acylglycerophosphocholines, which were predominantly elevated, with the largest increases in PC(P-16:0/20:4) and PC(O-22:0/20:4) (2.6-fold higher in OAG) (Cabrerizo et al., 2017). It also found that sphingomyelins (14 species, up to 2.75-fold increase) and cholesteryl esters (5 species, up to 2.44-fold increase) were also elevated, with class-level averages of +36% and +97% respectively; a decreased Cer/SM ratio (−71%) and increased SM+DG/Cer+PC ratio (+38%) pointed to altered sphingomyelin synthase and acid sphingomyelinase activity as a shared mechanism. Azbukina and colleagues later used targeted lipidomics of aqueous humor to identify a focused set of discriminating inflammatory lipid mediators in POAG, notably 12,13-DiHOME, AA, lyso-PAF, and oxidized linoleate species such as HODEs and KODEs, with 12,13-DiHOME and 13-KODE emerging as the most consistent discriminators across ANCOVA and PLS-DA analyses of a 24-patient POAG cohort (Azbukina et al., 2021).

More recent work incorporating plasma alongside AH has added another layer. In a cohort of 20 POAG and 20 angle-closure glaucoma (PACG) patients versus 20 cataract controls, phosphatidylcholines and lysophosphatidylcholines were elevated in AH of both groups (52.7% and 69.4% of PC species altered in POAG and PACG respectively), while sphingomyelins were markedly more prominent in PACG (85.7% of species elevated, versus 21.4% in POAG) and ceramides showed a similar disease-specific pattern (18.75% elevated in PACG versus 6.25% in POAG) (Gowtham et al., 2025). In PEXG, AH alterations have included cholesterol esters, phosphatidylcholines, triglycerides, and ceramides, all elevated relative to POAG, pseudoexfoliation syndrome, and control eyes, alongside an overall reduction in total AH lipid content across all three disease groups relative to controls (Collao et al., 2022).

### 4.2 Tears and ocular surface sampling

Tears are particularly attractive for translational biomarker development because collection is minimally invasive, repeatable, and compatible with longitudinal sampling (Chistyakov et al., 2020; Pieragostino et al., 2013; Mastropasqua et al., 2019; Panthi et al., 2019). Since many glaucoma patients use chronic topical medications, treatment confounding is substantial (Pieragostino et al., 2013). Tears nonetheless remain the most plausible substrate for a clinically deployable biomarker.

The most compelling translational signal so far is the observation that oxylipin disturbances identified in AH are mirrored in tears: in the same POAG cohort discussed above, 12,13-DiHOME, 9- and 13-HODE/KODE, arachidonic acid, and lyso-PAF distinguished glaucomatous from control tear fluid with the same direction of change as in AH (Azbukina et al., 2021; Chistyakov et al., 2020). This indicates that tear lipidomics captures aspects of intraocular inflammatory signaling, not merely surface inflammation. Future studies will need to separate disease signatures from medication effects by focusing on treatment-naïve cohorts, stratifying by drug class, or incorporating washout designs where ethically feasible.

### 4.3 Plasma and systemic lipidomics

Plasma biomarkers are appealing because blood sampling is routine and scalable. However, plasma lipidomics in glaucoma faces a major specificity problem: circulating lipids are influenced by diet, obesity, diabetes, lipid-lowering therapy, exercise, circadian rhythm, and many systemic inflammatory states (Ghosh and Nishtala, 2017). Even so, blood-based markers remain valuable if they complement ocular biomarkers rather than replace them.

Studies comparing AH and plasma suggest that ocular and systemic lipid signatures overlap only partially (Gowtham et al., 2025). Because the eye is protected by specialized barriers and governed by local metabolic circuits, ocular disease–associated biofluid signatures may be diluted or obscured in plasma. Despite this limited overlap, systemic oxidized lipid patterns such as hydroxylinoleate and hydroxyarachidonate isomers can still provide insight into the organism-wide pro-oxidant environment associated with glaucoma susceptibility or progression (Umeno et al., 2019). Plasma lipidomics will be most useful in multimodal models that integrate systemic risk, ocular imaging, and localized tear or AH data.

Age and biological sex are important sources of variation in the circulating lipidome. In the GOLDN cohort, age and sex were associated with numerous lipid species, but the direction of these relationships differed across lipid classes and between males and females. Women generally had higher concentrations of several phosphatidylcholines and sphingomyelins, whereas men had higher concentrations of other lipid species, including subsets of triglycerides. Age-by-sex interactions were particularly common among phosphatidylcholines, sphingomyelins, and triglycerides, indicating that age-related lipid changes do not occur uniformly in both sexes. For example, the study identified a sex-dependent age-related increase in choline-containing phospholipids, including phosphatidylcholines (Slade et al., 2021). Other population-based studies similarly found that ether phospholipids and lysophospholipids generally declined more strongly with age in men, whereas postmenopausal women had higher levels of several phosphatidylinositols, diacylglycerols, triacylglycerols, and alkyl-diacylglycerols but lower levels of ether phospholipids and lysophospholipids than premenopausal women (Beyene et al., 2020). In adults aged 56–100 years, plasma lipid levels generally declined with increasing age, particularly in men; however, women had higher levels of selected sphingomyelins and, after age 75, several docosahexaenoic acid-containing phospholipids (Wong et al., 2019). A systematic review of longitudinal studies also found that lysophosphatidylcholines, sphingomyelins, and phosphatidylethanolamines generally increased with age in women, whereas age-related phosphatidylcholine changes were inconsistent across studies (Mohammadzadeh et al., 2021). These findings demonstrate that age and sex influence both the abundance and longitudinal trajectories of specific lipid classes rather than producing a uniform increase or decrease across the entire lipidome.

These sources of lipidomic variation are particularly relevant to glaucoma because age and sex are not distributed equally across disease subtypes. The prevalence of POAG increases substantially with age and is modestly higher in men; a recent meta-analysis estimated that men had a 28% higher prevalence of POAG than women (Zhang et al., 2021). In contrast, primary angle-closure glaucoma (PACG) is more common in women and also increases markedly with age. In a meta-analysis of Asian populations, PACG prevalence increased from 0.21% at 40–49 years to 2.32% at 70 years or older (Cheng et al., 2014). These differences do not establish that age- or sex-dependent plasma lipid changes cause a particular glaucoma subtype. Rather, they demonstrate the potential for confounding: a study containing a greater proportion of older individuals, men with POAG, or women with PACG may identify apparent disease-associated lipid differences that partly reflect the demographic composition of the cohort. Accordingly, glaucoma lipidomic studies should match or adjust for age and sex, report results by glaucoma subtype, and assess sex-stratified and age-by-sex associations where sample size permits. Menopausal status and exogenous hormone exposure should also be considered when studying women. Because the available evidence derives predominantly from plasma or serum, whether similar age- and sex-dependent patterns occur in aqueous humor, tears, trabecular meshwork, or retinal tissue remains insufficiently characterized.

### 4.4 Retina and optic nerve

Unlike aqueous humor, tears, and plasma, retinal and optic nerve findings discussed in this section derive primarily from tissue-based experimental or *ex vivo* studies rather than routinely sampled human biofluids. Direct human retinal and optic nerve lipidomics in glaucoma are limited by tissue availability, but experimental studies show that injury and regeneration are accompanied by broad lipid remodeling (Simon et al., 2021; Funke et al., 2016). These data provide the mechanistic bridge often missing from clinical fluid studies. For example, if an AH biomarker correlates with disease severity but no retinal mechanism is known, its role remains ambiguous. By contrast, evidence of parallel changes in retinal oxylipins, sphingolipids, or inflammatory lipid networks strengthens causal interpretation.

Animal and ex-vivo studies indicate that retinal injury can alter phospholipid classes, sphingolipid balance, oxylipin production, and sterol metabolism (Simon et al., 2021; Mondal and Mandal, 2019; Ahmad et al., 2024). Optic nerve regeneration studies likewise suggest that lipid remodeling is not merely a marker of degeneration; it also participates in repair programs (Trzeciecka et al., 2019). Importantly, retinal lipid metabolism is also highly cell specific, and bulk lipidomic measurements may obscure distinct metabolic responses occurring within individual retinal cell populations. RGCs, astrocytes, microglia, Müller glia, and vascular cells differ in their capacity for lipid synthesis, uptake, oxidation, storage, and remodeling, meaning that the same lipid class may have different biological consequences depending on its cellular source and metabolic context. This distinction is particularly relevant in glaucoma, where lipid signaling can simultaneously influence neuronal survival, glial activation, inflammation, and vascular homeostasis. For example, astrocyte-derived pro-resolving mediators such as LXA4/LXB4 can promote RGC survival, whereas ceramide accumulation within stressed neuronal or glial compartments may promote mitochondrial dysfunction and apoptosis (Edwards et al., 2020; Zhou et al., 2026; Jager et al., 1993; Chistyakov et al., 2020). Thus, future glaucoma lipidomics should move beyond bulk measurements toward cell-resolved or spatially resolved approaches, including single-cell transcriptomic or lipidomic integration, to determine which cell populations generate or respond to disease-associated lipid signatures. Future lipid biomarkers may predict not only injury burden but also recovery potential or therapeutic responsiveness.

### 4.5 Biomarker utility: detection, staging, prognosis, and treatment response

For translation, the question is not whether lipids differ between glaucoma and controls but whether they can improve diagnosis or management.

First, compartment-specific lipidomic signatures may aid in early detection or risk stratification, especially when combined with imaging and functional measures. Second, they support subtype classification; for example, distinguishing POAG, angle-closure glaucoma, and PEXG based on partially distinct lipid networks (Gowtham et al., 2025; Collao et al., 2022). Third, they function as progression markers if certain mediators correlate with structural or visual field deterioration over time. Fourth, they serve as pharmacodynamic markers that indicate target engagement for new therapies such as sEH inhibitors or cannabinoid modulators.

At present, evidence is strongest for biomarker discovery and weakest for prognostic or predictive use. Most studies remain cross-sectional and relatively small. Translation will require multicenter validation, standardized analytics, repeated sampling, and direct linkage to clinically meaningful endpoints.

### 4.6 Cross-compartment lipid trafficking

The five sampling compartments described above are not equally accessible to one another, and the degree of overlap between any two reflects specific anatomical and physiological constraints rather than a uniform leakiness of the eye. Plasma and AH are separated by the blood-aqueous barrier, formed by tight junctions of the non-pigmented ciliary epithelium and the iris vascular endothelium; these are “leaky-type” junctions that permit some pressure-dependent solute exchange, in contrast to the “non-leaky” tight junctions of the blood-retinal barrier at the retinal pigment epithelium and retinal vascular endothelium (Cunha-Vaz, 1979). This asymmetry helps explain why AH and plasma lipid signatures overlap only partially: AH composition is shaped substantially by local secretion and local eicosanoid synthesis in the ciliary body and trabecular meshwork; for example, mechanosensitive TRPV4 activation in trabecular meshwork cells stimulates local arachidonic acid release and prostaglandin production independent of circulating lipid pools (Uchida et al., 2021), whereas plasma lipids primarily reflect systemic metabolic processes, including dietary intake, hepatic lipid metabolism, and inflammatory status, rather than local ocular lipid regulation (Quehenberger and Dennis, 2011); transport of circulating lipids into AH is restricted by the blood-aqueous barrier and occurs only to a limited extent (Cunha-Vaz, 1979). AH and tears show closer correspondence for a related but distinct reason: both contain overlapping lipid mediator signatures generated through shared COX-, LOX-, and CYP-derived oxylipin pathways within the anterior segment, consistent with the similarity in oxylipin profiles reported between AH and tear fluid (Chistyakov et al., 2020). Although the vitreous cavity and blood–retinal barrier limit exchange between the posterior and anterior segments, AH may still capture a subset of molecular alterations originating from the retina and vitreous. Wilson et al. demonstrated substantial overlap between AH and vitreous protein profiles in patients with retinal diseases: 812 proteins were detected in both compartments, and 490 showed significant correlations with correlation coefficients greater than 0.5 (Wilson et al., 2023). These findings support the potential use of AH as a clinically accessible surrogate for selected posterior-segment biomarkers. However, this evidence derives from proteomic analysis, and the extent to which lipid species demonstrate comparable correspondence between AH, vitreous, retina, and optic nerve remains unknown. Accordingly, AH lipidomics may provide information about posterior-segment disease processes, but matched AH–vitreous lipidomic studies are needed to determine which lipid classes reliably reflect retinal or optic nerve biology.

### 4.7 Cross-study concordance and discordance

Direct comparison across the four AH lipidomic studies reveals both convergent and conflicting signals, summarized in Table 2. Sphingomyelins show the most consistent direction of change: Cabrerizo reported elevations up to 2.75-fold in OAG (Cabrerizo et al., 2017), Collao found sphingomyelins among the classes elevated in PEXG relative to control (Collao et al., 2022), and Gowtham reported sphingomyelin elevation in both POAG and PACG, more pronounced in PACG (Gowtham et al., 2025). Azbukina’s targeted oxylipin panel did not include structural sphingomyelins, so its absence from this comparison reflects a difference in analytical scope rather than a contradictory finding (Azbukina et al., 2021).

**TABLE 2:** Structured evidence table of glaucoma lipidomic biomarker studies. One row per study/matrix. AH = aqueous humor; PG = prostaglandin; CAI = carbonic anhydrase inhibitor; FC = fold change.

First author, year (ref)	Matrix	n cases/n controls	Glaucoma subtype	Treatment status	Platform (targeted/untargeted)	Key analytes	Direction and fold change	Statistical correction applied	Independent replication
Azbukina et al., 2021	AH	24 POAG/14 cataract controls	POAG, stages II–III (IOP-stratified subgroups)	On treatment (PG analogs, beta-blockers, CAI, alpha-agonists); treatment effect explicitly tested — none found	Targeted UPLC-MS/MS (22 pre-specified signaling lipids)	12,13-DiHOME, 13-KODE, lyso-PAF, AA, 9-HODE	12,13-DiHOME ↑, 13-KODE ↑ (both stage-dependent); lyso-PAF ↑ (PLS-DA only); AA ↑; 9-HODE ↓. Concentrations reported, not fold-change	ANCOVA + holm–Bonferroni (p < 0.05); PLS-DA VIP>1.5 (uncorrected)	No
Azbukina et al., 2021	Tear fluid	24 POAG/14 cataract controls (same patients)	POAG, stages II–III	Same as above; treatment effect tested — none found	Targeted UPLC-MS/MS (same 22-lipid panel)	12,13-DiHOME, 9-/13-HODE/KODE, AA, lyso-PAF, DHA	Same direction as AH for shared analytes; DHA ↓ (TF-specific)	Same as above	No; cross-compartment concordance within same cohort
Gowtham et al., 2025	AH	20 POAG +20 PACG/20 cataract controls (AH subset n = 11/group)	POAG and PACG (separate arms)	On treatment (90%/75% on PG analogues, POAG/PACG); not tested for confounding	Untargeted HRMS, LipidSearch (301 AH species)	PC, LPC ↑ both; SM, ceramides ↑ (PACG-predominant); TG, DG ↓ both	PC/LPC ↑ in 52.7%–69.4% of species (log_2_FC ≥ 1); SM ↑ in 85.7% PACG vs. 21.4% POAG; ceramides: log_2_FC −5.05/−3.82 (POAG, ↓) vs. ≥ 1.58 (PACG, ↑)	log_2_FC ≥ 1/≤−1 + p < 0.05 + VIP>1 (MetaboAnalyst); no FDR correction across species	No
Gowtham et al., 2025	Plasma	20/20/20 (n = 20 per group)	POAG and PACG	Same as AH arm above	Untargeted HRMS (1762 species)	No class shifted >20%; 47/53 individual species significant (POAG/PACG)	No dominant class-level signal; individual species scattered ↑/↓	Same criteria as AH arm	No
Collao et al., 2022	AH	19 POAG, 9 PEX, 14 PEXG/23 controls	POAG, PEX, PEXG (compared pairwise and to control)	Not stated in available text	Untargeted UHPLC-MS (489 species, 26 classes)	ChE, PC, TG, ceramides ↑ in PEXG; CerG2GNAc1(d34:1) ↓ in PEX and PEXG	Total lipid content ↓ in all disease groups vs. control (p < 0.001); fold-changes not tabulated numerically in available text	ANOVA-based; ML classification (86%/71%/86% accuracy); no explicit FDR	No
Cabrerizo et al., 2017	AH	10 OAG/10 controls	OAG (generic, not substaged)	On treatment; 7-day washout before surgery	Untargeted UHPLC-ToF-MS (110 species screened)	SM (14 species) ↑, ChoE (5 species) ↑, DAPC/MEMAPC (19 of 37 sig. Species) mostly ↑, 2 PC species ↓	SM up to 2.75-fold (SM d18:2/20:0); ChoE up to 2.44-fold (ChoE 20:4); PC(P-16:0/20:4)/PC(O-22:0/20:4) ∼2.6-fold ↑; PC(40:1) 0.22-fold, PC(O-38:4) 0.23-fold ↓	Unpaired Student/Welch t-test, p < 0.05; uncorrected for multiple comparisons (37/110 tested)	No; directional class-level concordance with gowtham for SM

Ceramides, by contrast, show genuine discordance. Gowtham reported two specific ceramide species decreased in POAG (Cer(d18:2/24:1), Cer(d18:1/24:1); log_2_FC −5.05 and −3.82) while ceramides broadly increased in PACG (Gowtham et al., 2025). Cabrerizo’s ceramide signal was not isolated from its sphingomyelin-driven findings and therefore neither confirms nor contradicts this pattern. Given that the two studies used different platforms (semi-targeted versus untargeted discovery) and neither corrected for multiple comparisons, species-level ceramide direction should be treated as provisional pending independent replication.

Phosphatidylcholines and lysophosphatidylcholines showed broad net elevation across all three studies that measured them: Cabrerizo reported predominantly increased diacyl- and 1-ether,2-acylglycerophosphocholines (Cabrerizo et al., 2017), Gowtham found phosphatidylcholines elevated in roughly 53%–69% of species measured and lysophosphatidylcholines elevated in both POAG and PACG (Gowtham et al., 2025), and Collao reported phosphatidylcholines among the classes elevated in PEXG (Collao et al., 2022). However, Cabrerizo also identified two specific phosphatidylcholine species that were decreased (PC(40:1), PC(O-38:4)), illustrating that class-level predominantly increased summaries can obscure species moving in the opposite direction (Cabrerizo et al., 2017). Because none of the three studies used matching nomenclature or overlapping platforms, individual phosphatidylcholine species cannot be cross-matched between datasets as reported.

Total lipid content and triglycerides showed concordant directionality at the class level: Collao reported reduced total AH lipid content across all disease groups relative to control (Collao et al., 2022), and Gowtham reported triglycerides specifically decreased in both POAG and PACG (Gowtham et al., 2025). These findings agree in direction but used different control definitions and platforms and were not designed for direct quantitative comparison. Cholesterol esters showed similar cross-study agreement: Cabrerizo reported increases up to 2.44-fold in OAG (Cabrerizo et al., 2017), and Collao reported cholesterol esters elevated in PEXG relative to all other groups, with ChE(20:5) the most enriched species (Collao et al., 2022). Because these findings span different glaucoma subtypes and platforms, they represent consistent supporting evidence rather than strict replication.

Azbukina’s targeted oxylipin panel (12,13-DiHOME, 9-/13-HODE/KODE, lyso-PAF, arachidonic acid) has no counterpart in the other three AH studies, none of which included a targeted oxylipin analysis (Azbukina et al., 2021). This represents a difference in panel design rather than a failed replication, and highlights a broader gap in the current literature: oxylipin-level findings remain unconfirmed by any independent cohort or overlapping platform.

## 5 Therapeutic horizons

The therapeutic approaches discussed below differ substantially in their level of validation and should not be interpreted as equally ready for clinical use. They can be broadly divided into three groups: therapies already used clinically in glaucoma, particularly prostaglandin-based treatments; interventions with glaucoma-specific preclinical or early human evidence, including autotaxin–lysophosphatidic acid signaling, endocannabinoid modulation, and specialized pro-resolving mediators; and biologically plausible strategies that remain insufficiently validated in glaucoma, including soluble epoxide hydrolase inhibition, platelet-activating factor targeting, and sphingolipid pathway modulation.

### 5.1 Therapies already used clinically: prostanoid pharmacology

Prostaglandin analogues are established clinical therapies for glaucoma and ocular hypertension and are among the most effective ocular hypotensives, but their mechanisms extend beyond FP receptor activation and uveoscleral outflow (Tam et al., 2013; Weinreb et al., 2020).

Bimatoprost SR/Durysta reframes prostanoid therapy as both drug delivery and tissue remodeling. IOP lowering can persist beyond the expected period of active drug exposure, suggesting sustained remodeling of outflow tissues (Medeiros et al., 2020; Stamer et al., 2023). Matrix metalloproteinase-mediated extracellular matrix modification in the ciliary muscle, TM, Schlemm canal, and adjacent drainage tissues likely contributes to this durability (Weinreb et al., 2020; Stamer et al., 2023).

Latanoprostene bunod extends prostanoid pharmacology by combining FP receptor activity with nitric oxide donation. After ocular metabolism, it yields latanoprost acid, which promotes prostaglandin-mediated outflow, and a nitric oxide–donating moiety, which relaxes the TM and Schlemm canal to enhance conventional outflow (Impagnatiello et al., 2019; Cavet and DeCory, 2018).

Prostaglandin analogues also influence ocular lymphatic drainage. Latanoprost stimulates lymphatic drainage from the eye *in vivo*, lowering IOP partly through a uveolymphatic pathway, and can induce conjunctival lymphatic vessel development in rabbits (Mathew and Sivak, 2024; Tam et al., 2013; Kim et al., 2017; Ma et al., 2021). These findings support lymphatic and lymphatic-like outflow as under-recognized contributors to prostaglandin-mediated IOP lowering.

### 5.2 Glaucoma-relevant, investigational

The endocannabinoid system represents a glaucoma-relevant investigational therapeutic target, supported by experimental studies and limited human clinical evidence (Gagliano et al., 2011). Older clinical work focused on cannabis and short-lived IOP reduction (Jay and Green, 1983; Green, 1979). The modern translational narrative is more sophisticated. It considers receptor subtype selectivity, enzyme inhibition, local delivery, allosteric modulation, and multi-target effects on IOP, inflammation, blood flow, and neuroprotection (Edwards et al., 2014b).

Several strategies are conceivable. One is direct activation of ocular cannabinoid pathways using topical or sustained-release agonists. Another is inhibition of degradative enzymes such as fatty acid amide hydrolase or monoacylglycerol lipase to raise endogenous ligand levels locally. A third is exploitation of non-classical cannabinoid-sensitive receptors such as GPR18 or TRPV1. Because cannabinoid signaling intersects with pain, inflammation, vascular tone, and neuronal survival, it may be especially useful as part of combination therapy in patients progressing despite adequate IOP control. None of these approaches is currently an established glaucoma therapy.

SPMs offer a distinct strategy: restoring endogenous resolution programs rather than suppressing inflammatory initiation. In chronic glaucoma, this approach is more physiologic. Lipoxin-based or analogous therapies could reduce glial overactivation, improve retinal resilience, and perhaps preserve synaptic integrity. Major development challenges include molecular stability, receptor specificity, local delivery, and the difficulty of defining appropriate treatment windows in slowly progressive disease. Still, among pressure-independent strategies, SPM biology is one of the most compelling because it aligns closely with the idea of glaucoma as a chronic disorder of unresolved stress. Mathew et al. extend this concept by showing that latanoprost itself induces an anterior chamber AA/LXA_4_ circuit in glaucoma patients, suggesting that some conventional IOP-lowering drugs may also engage endogenous resolution pathways (Mathew and Sivak, 2024; Mathew et al., 2026).

### 5.3 Biologically plausible, not yet glaucoma-validated

sEH converts epoxy-fatty acids into corresponding diols such as DiHOMEs (Hildreth et al., 2020; Marowsky et al., 2017; Park and Corson, 2019). In several organ systems, epoxy-fatty acids are considered beneficial or protective, while excess diol formation is maladaptive. 12,13-DiHOME in glaucoma AH signatures makes this pathway attractive (Azbukina et al., 2021). sEH inhibition has already been proposed for ocular diseases more broadly (Park and Corson, 2019). In glaucoma, such inhibition could theoretically rebalance inflammatory tone, vascular regulation, and oxidative lipid signaling. Because sEH sits downstream of multiple polyunsaturated fatty acid pathways, it could offer broader network correction than targeting a single prostaglandin receptor. However, sEH inhibition has not yet been validated as a glaucoma treatment, and its current rationale is based primarily on lipidomic associations and broader ocular evidence (Park and Corson, 2019).

Given the evidence for phospholipase A2 activity in ocular tissues (Rönkkö et al., 2007; Helin et al., 2008), and the presence of lyso-PAF-related disturbances in glaucoma, upstream modulation of phospholipid hydrolysis deserves renewed consideration (Azbukina et al., 2021; Chistyakov et al., 2020). However, this strategy is complicated by the centrality of phospholipase activity to normal cellular homeostasis. A more feasible near-term approach is selective interference with PAF signaling or downstream inflammatory consequences. Older preclinical data showing that PAF antagonism can affect ocular hypertension (Jager et al., 1993) provide proof of principle, but modern translation would require better receptor pharmacology, local delivery systems, and clearer patient stratification. PAF-directed therapy therefore remains a preclinical strategy rather than a validated glaucoma treatment.

Ceramides are frequently linked to apoptosis and inflammatory stress; hence, therapies that reduce ceramide accumulation or shift signaling toward less harmful sphingolipid states could be beneficial (Aljohani et al., 2013; Edwards et al., 2014a; Simon et al., 2021; Mondal and Mandal, 2019; Edwards et al., 2014b). Potential strategies include inhibiting ceramide-generating enzymes, altering sphingomyelin turnover, or modulating S1P receptor signaling. Yet sphingolipid pathways are highly pleiotropic and systemically important, so ocular-specific delivery or tissue-restricted targeting will be critical. Even if direct therapeutic manipulation proves difficult, sphingolipid signatures may still be useful for identifying patients with more inflammatory or fibrotic glaucoma phenotypes. However, direct pharmacological manipulation of sphingolipid pathways has not yet been validated as an effective glaucoma treatment.

### 5.4 Combination therapy and precision medicine

The future of glaucoma treatment is unlikely to rest on a single new lipid drug. More plausibly, lipid therapeutics will complement existing pressure-lowering regimens. For example, a prostaglandin analogue could eventually be paired with a neuroprotective cannabinoid modulator, an sEH inhibitor, or an SPM analogue. Biomarkers could guide this process by identifying patients with dominant oxidative, ceramide-rich, or inflammatory-resolving deficiencies. Lipidomics could support precision medicine both by providing targets and by helping match them to patient subgroups.

## 6 Challenges and path forward

### 6.1 How current glaucoma therapies distort lipidomic readouts

A major challenge in interpreting glaucoma lipidomics is distinguishing disease-associated lipid remodeling from pharmacodynamic effects of IOP-lowering therapy. This issue is particularly important for topical prostaglandin analogues (PGAs), because these agents directly engage endogenous lipid signaling pathways and therefore have the potential to modify the same lipid mediators being investigated as disease biomarkers. Recent lipidomic analysis of AH from glaucoma patients identified increased arachidonic acid (AA) and lipoxin A4 (LXA4), and subsequent experiments demonstrated that this signature was induced by latanoprost rather than elevated IOP alone. Latanoprost increased expression of lipid-metabolizing enzymes in human trabecular meshwork cells and increased AA and LXA4 production, providing direct evidence that a clinically prescribed glaucoma therapy can generate a lipidomic signature that could otherwise be misinterpreted as disease-associated biology (Azbukina et al., 2021; Mathew et al., 2026).

Importantly, the potential for treatment-related distortion is not necessarily restricted to prostaglandin analogues. In the same experimental study, latanoprost, timolol, dorzolamide, and brimonidine were evaluated in human trabecular meshwork cells; however, the most consistent and pronounced induction of the AA/LXA4 pathway was observed with latanoprost.37 Thus, while other topical therapies may plausibly influence the ocular lipid environment through their effects on AH dynamics and cellular signaling, direct evidence for reproducible alterations in specific lipidomic species remains substantially less developed than for PGAs.

Treatment exposure should therefore be considered a primary design variable in glaucoma lipidomic studies rather than a secondary clinical covariate. Treatment-naïve cohorts are preferable for discovery studies because they provide the clearest opportunity to distinguish disease-associated lipid abnormalities from pharmacodynamic signatures. When treated populations are studied, medication class, duration of exposure, number of agents, dosing frequency, and time since the most recent administration should be recorded and incorporated into the analysis. Where clinically and ethically feasible, paired pre- and post-treatment sampling could further distinguish disease-associated lipid signatures from treatment-induced changes. This is particularly important for aqueous humor, where the measured lipid profile represents a local biological environment influenced by disease, mechanical stress, topical therapy, and inflammatory signaling. Longitudinal studies incorporating treatment-naïve baseline samples and repeated sampling after treatment initiation will ultimately be required to determine whether candidate lipid signatures represent stable features of glaucoma, markers of disease progression, or pharmacodynamic responses to therapy.

### 6.2 Methodological and biological limitations of ocular lipidomics

Despite growing evidence that lipid metabolism is altered in glaucoma, ocular lipidomics remains far from clinical translation. A central limitation is cohort design. Most studies rely on small samples collected opportunistically during surgery, often comparing glaucoma patients with cataract controls. This approach is practical because aqueous humor is difficult to obtain, but it introduces substantial bias: surgical cohorts are typically older, heavily treated, and affected by coexisting ocular disease. As a result, lipid differences reflect glaucoma biology, cataract-associated ageing, chronic medication exposure, or selection for surgery rather than disease-specific mechanisms. Future studies will require larger, multicentre cohorts with harmonized inclusion criteria, standardized phenotyping, and appropriate control groups.

Several methodological limitations constrain interpretation of the existing ocular lipidomics literature and should inform future study design. First, reliance on surgical cohorts introduces selection bias, as patients undergoing incisional glaucoma surgery represent a more advanced or refractory disease subset than the broader glaucoma population, limiting generalizability to earlier-stage or medically controlled disease. Second, the common practice of using cataract surgery patients as controls is imperfect, since cataract itself is associated with lens-derived oxidative stress and age-related metabolic changes that may independently alter aqueous humor lipid composition, potentially attenuating or confounding true case-control differences (Lillo et al., 2022). Third, aging is a significant unaddressed confounder: lipid metabolism, membrane composition, and oxidative lipid burden change substantially across the lifespan, and glaucoma cohorts are frequently older and imperfectly age-matched to controls, making it difficult to separate disease-specific signatures from age-related lipidomic drift (Huang et al., 2025). Fourth, diet and systemic metabolic status—including obesity, dyslipidemia, diabetes, and omega-3/omega-6 fatty acid intake—are rarely captured in ocular lipidomic studies despite their known capacity to shape circulating and potentially local lipid mediator pools, as plasma oxylipin profiles (including arachidonic acid–derived HETEs and DiHETrEs) have been shown to correlate with adiposity and insulin resistance in humans, raising the possibility that unmeasured metabolic heterogeneity contributes to inconsistent findings across cohorts (Jurado-Fasoli et al., 2023). Fish-oil supplementation is particularly relevant because its principal omega-3 fatty acids, EPA and DHA can alter the substrates available for oxylipin and specialized pro-resolving mediator synthesis. Preclinical studies have reported that dietary omega-3 supplementation increases aqueous outflow, lowers IOP, and reduces retinal inflammation and RGC loss in experimental glaucoma models (Kalogerou et al., 2018; Nguyen et al., 2007). Limited human evidence is also suggestive: 3 months of approximately 1,500 mg/day combined EPA and DHA reduced IOP by approximately 8% in normotensive adults, while a 6-month open-label randomized trial of DHA combined with antioxidants in pseudoexfoliation glaucoma reported reductions in oxidative-stress markers and IOP (Romeo et al., 2018; Downie and Vingrys, 2018). However, the former study did not enrol patients with glaucoma, and the latter used a multicomponent supplement, preventing the observed effects from being attributed specifically to DHA or fish oil. Fish-oil supplementation should therefore be regarded as biologically plausible but not an established treatment for glaucoma. Future trials should evaluate standardized EPA and DHA formulations in defined glaucoma subtypes and assess IOP, ocular blood flow, structural progression, visual-field outcomes, and ocular lipidomic changes. A recent review provides a detailed synthesis of the proposed IOP-lowering, vascular, anti-inflammatory, and antioxidative mechanisms of fish oil in glaucoma (Ye et al., 2023). Finally, pre-analytical sample handling remains inadequately standardized across studies; variability in collection technique, time-to-freezing, storage temperature, freeze-thaw cycling, and anticoagulant or preservative exposure can introduce lipid oxidation and degradation artifacts that are difficult to distinguish from genuine biological signal, particularly in the low-volume ocular samples characteristic of this field (Anton et al., 2015). Together, these factors underscore the need for prospective, age- and metabolically-matched cohorts with standardized pre-analytical protocols before candidate lipid signatures can be considered robust or disease-specific.

Medication confounding is an equally important obstacle. Most clinically diagnosed glaucoma patients are already receiving topical therapy, and prostaglandin analogues, beta-blockers, carbonic anhydrase inhibitors, rho-kinase inhibitors, preservatives, and ocular surface inflammation reshape tear or aqueous humor lipid profiles. Future work should separate three related questions: which lipid signatures are present before treatment, which change after therapy, and which persist despite adequate pressure lowering. Treatment-naïve cohorts, pre–post intervention designs, medication washout studies where ethical, and careful adjustment for drug class, duration, preservative exposure, and ocular surface status will be essential.

The recent Mathew et al. AH study provides a concrete example of this problem: a glaucoma-associated AA/LXA4 signature initially appeared disease related, but mechanistic experiments implicated latanoprost-induced lipid remodeling (Mathew and Sivak, 2024; Mathew et al., 2026). This illustrates why future ocular lipidomic studies must distinguish untreated disease biology from treatment-induced pharmacodynamic signatures.

Analytical reproducibility is another prerequisite for progress. Lipidomics is highly sensitive to pre-analytical and analytical variation, especially in low-volume ocular samples. Collection method, storage temperature, oxidation, freeze–thaw cycles, extraction chemistry, ion suppression, adduct formation, and internal standard selection can all affect results. Therefore, standard operating procedures are not merely technical details but core requirements for biological interpretation. The field should move from broad untargeted discovery toward validated targeted panels using isotope-labelled standards, predefined quality-control metrics, and independent replication across laboratories. Candidate panels should prioritize reproducible biologic domains, including arachidonic acid–derived oxylipins, HODE/KODE species, DiHOME-related metabolites, phosphatidylcholine and lysophosphatidylcholine species, and ceramide/sphingomyelin pathways.

A further challenge is disease heterogeneity. Glaucoma is a complex disease: primary open-angle glaucoma, normal-tension glaucoma, PEXG, and angle-closure glaucoma likely share some pathways while differing in pressure dependence, vascular dysregulation, extracellular matrix remodelling, oxidative stress, and inflammatory tone. Even within primary open-angle glaucoma, lipid signatures distinguish patients with rapid structural progression, ocular surface inflammation, metabolic vulnerability, or pressure-independent neurodegeneration. Future studies should therefore prespecify clinical use-cases—diagnosis, progression prediction, treatment selection, or pharmacodynamic monitoring—rather than simply reporting cross-sectional differences.

Sex is another potentially important source of biological heterogeneity that has received comparatively little attention in glaucoma lipidomics. Glaucoma subtypes differ in their sex distributions and may also exhibit sex-dependent differences in vascular, hormonal, metabolic, and inflammatory pathways that could influence lipid metabolism and disease susceptibility. Epidemiologic studies have reported differences in the relative prevalence and clinical characteristics of glaucoma subtypes between men and women, with primary angle-closure glaucoma showing a particularly strong female predominance, whereas primary open-angle glaucoma is more common overall in men in many populations (Cheng et al., 2013; Kapetanakis et al., 2016). These differences may be relevant to lipid biology because sex hormones can influence lipid synthesis, transport, oxidation, and inflammatory signaling, potentially contributing to differences in disease phenotype or progression (Zore et al., 2018; Wang et al., 2011). Accordingly, future lipidomic studies should prespecify sex as a biological variable and evaluate sex-by-subtype interactions rather than simply adjusting for sex as a covariate. Sex-stratified analyses may help determine whether candidate lipid signatures are broadly associated with glaucoma or instead reflect biologically distinct pathways operating preferentially in particular patient groups.

The most useful path forward is an integrated, longitudinal research agenda. Ocular and systemic samples should be studied together to distinguish local from whole-body lipid signatures. Lipidomics should be paired with optical coherence tomography, OCT-angiography, visual fields, IOP profiling, corneal biomechanics, medication history, and, where possible, transcriptomic, proteomic, genetic, and mechanistic model data. Network-level measures, such as epoxy-to-diol balance, ceramide-to-sphingomyelin balance, or pro-inflammatory-to-pro-resolving mediator balance may prove more stable than individual analytes. In the near term, lipidomics may be valuable for stratifying patients and enriching trials for biologically defined subgroups. This would allow lipidomic discovery to move beyond association and toward clinically actionable glaucoma biology.

### 6.3 Multi-omics integration, AI-assisted biomarker discovery, and clinical validation

Single-omics discovery is unlikely to fully resolve the translational barriers described above, and emerging frameworks increasingly emphasize integration across molecular layers. A recent multi-omics framework for glaucoma proposes integrating genomic, transcriptomic, epigenomic, proteomic, metabolomic (including lipidomic), microbiome, and phenotypic data into a multimodal probabilistic model to estimate disease state and future progression risk, rather than interpreting each molecular layer independently (Li et al., 2026). For lipid mediators specifically, multi-omics integration provides a framework to distinguish lipid signals that are more likely to represent upstream biological drivers, such as those influenced by genetic risk variants (e.g., ABCA1), from secondary alterations arising downstream of tissue injury or cellular stress responses captured through transcriptomic and proteomic profiling of the same biological system.

Artificial intelligence and machine-learning approaches provide the computational framework required to analyze these high-dimensional datasets, where the complexity and interactions across multiple molecular layers exceed the capacity of conventional univariate approaches (Hasin et al., 2017). Reviews of AI in glaucoma demonstrate applications across diagnosis, progression monitoring, treatment planning, and surgical outcome prediction, while also emphasizing persistent limitations related to external validation, standardization, interpretability, and generalizability (Mishra et al., 2025; Ji et al., 2024; Kailani et al., 2025). A concrete precedent for lipid-adjacent biomarker work exists in angle-closure glaucoma, where a machine-learning model applied to targeted serum metabolomics identified androstenedione as a diagnostic biomarker with strong discriminative performance across multicenter discovery and validation cohorts, while higher baseline levels were associated with faster 2-year visual field progression (Li et al., 2024).

Translational lipidomics will require a similar progression from exploratory molecular signatures to reproducible and clinically actionable biomarkers. Candidate lipid signatures should therefore be evaluated using standardized sampling and analytical protocols, targeted quantitative assays, multicenter recruitment, independent external validation cohorts, and predefined clinically meaningful endpoints such as disease progression or treatment response. Integration of lipidomic measurements with established clinical variables and other molecular layers should also determine whether lipid biomarkers provide incremental predictive value beyond existing clinical models. Such a framework could be applied to candidate glaucoma pathways including oxidized linoleates, ATX–LPA signaling, and ceramide/sphingomyelin metabolism. Lipidomics is therefore best viewed as one component of a broader multi-omics framework, with clinical utility ultimately dependent on prospective validation and demonstration of added value beyond established diagnostic and prognostic measures.

## 7 Conclusion

Lipid biology links pressure stress, trabecular meshwork dysfunction, oxidative injury, vascular dysregulation, glial activation, and retinal ganglion cell death. However, the field does not need more isolated cross-sectional lipid differences; it needs clearer translational priorities.

Several study-design elements should be prioritized in the next phase of research. The highest priority is treatment-naïve cohorts: most reported lipid signatures to date cannot be confidently separated from medication exposure (Section 6.1), and sampling before treatment initiation, or using pre/post-treatment designs, is the fix that every other design improvement depends on, since without it the confound simply propagates downstream. Closely related is longitudinal sampling, because cross-sectional comparisons cannot distinguish stable trait markers from state-dependent or progression-associated signals; repeated sampling over time is required to determine whether a lipid signature predicts progression, tracks with treatment response, or is simply present at diagnosis. A third priority is matched ocular and systemic profiling. Because AH and plasma lipid signatures overlap only partially (Section 4.6), studies that sample only one compartment cannot determine whether a candidate marker reflects local ocular pathology, systemic metabolic status, or both, and paired sampling is necessary to assign a signal to its correct biological source. Fourth, the field needs targeted validation panels: untargeted discovery lipidomics has generated candidate markers faster than it has validated them, and moving toward predefined, isotope-labelled, quality-controlled targeted panels is what will let independent laboratories actually replicate findings. Finally, subtype-stratified glaucoma cohorts are essential, since POAG, angle-closure glaucoma, PEXG, and normal-tension glaucoma show partially distinct lipid networks (Section 4.5), and pooling subtypes in discovery cohorts risks averaging away subtype-specific signals that would otherwise be clinically actionable.

Beyond study design, therapeutic strategy also needs explicit prioritization. Lipid-directed approaches, soluble epoxide hydrolase inhibition, ATX–LPA pathway modulation, specialized pro-resolving mediators, and endocannabinoid signaling should be ranked by mechanistic plausibility, druggability, and glaucoma-specific, rather than only broader vascular or inflammatory, validation. Soluble epoxide hydrolase inhibition illustrates this tension well: it is mechanistically attractive because it connects vascular regulation, inflammation, oxidative stress, and neuroprotection through a single defined pathway, but its glaucoma-specific evidence base remains less mature than its rationale in other organ systems. Tear lipidomics, despite AH being mechanistically closer to anterior-segment pathology, may prove more clinically usable than AH lipidomics precisely because it can be sampled repeatedly and non-invasively, which AH cannot.

Within the reproducible biologic domains discussed throughout this review, four candidates stand out as most ready for targeted validation given their current evidence base. The 12,13-DiHOME/oxidized-linoleate axis (including HODE and KODE species) has shown concordant alterations in both AH and tears within targeted lipidomic studies, giving it cross-compartment reproducibility that few other candidates share, and it maps directly onto a druggable pathway through soluble epoxide hydrolase inhibition, making it a natural first target for a combined biomarker-and-therapeutic validation program. The autotaxin–LPA axis is similarly well positioned: it has been replicated across independent human AH cohorts, shows significant association with IOP, and already has pharmacologic tools (ATX inhibitors) available for mechanistic follow-up, so validation here would test a biomarker and a therapeutic hypothesis simultaneously. Phosphatidylcholine and lysophosphatidylcholine species have been reported across multiple AH cohorts spanning open-angle, angle-closure, and PEXG, and their consistent, if variable, presence across subtypes makes them a strong candidate for subtype-stratified validation specifically. The ceramide/sphingomyelin pathway rounds out this set, given its reproducible elevation in AH, particular prominence in angle-closure disease, and an existing therapeutic rationale in sphingolipid-rheostat modulation. By contrast, the AA/LXA4 circuit, while mechanistically well characterized, is better positioned for near-term validation as a pharmacodynamic marker of prostaglandin analogue engagement than as a disease biomarker, given the treatment-confounding evidence discussed in Section 6.1.

The most realistic near-term goal is not universal lipidomic screening, but patient stratification. The clinical need for such stratification is particularly evident in angle-closure disease, where only a subset of primary angle-closure suspects progresses and current anatomical predictors remain insufficient for individualized molecular risk assessment (Ji et al., 2025). Longitudinal tear and plasma lipidomics, paired with OCT, visual fields, IOP history, treatment exposure, and mechanistic validation, could identify biologically distinct subgroups and help match patients to targeted interventions. Executed with the priorities and candidate pathways above, this would move lipidomics in glaucoma beyond descriptive biomarker discovery and toward mechanism-guided therapy.
